# Culture substrate stiffness impacts human myoblast contractility-dependent proliferation and nuclear envelope wrinkling

**DOI:** 10.1242/jcs.261666

**Published:** 2024-03-27

**Authors:** Jo Nguyen, Lu Wang, Wen Lei, Yechen Hu, Nitya Gulati, Carolina Chavez-Madero, Henry Ahn, Howard J. Ginsberg, Roman Krawetz, Matthias Brandt, Timo Betz, Penney M. Gilbert

**Affiliations:** ^1^ Institute of Biomedical Engineering, University of Toronto, Toronto, ON, M5S 3E2, Canada; ^2^ Donnelly Centre, University of Toronto, Toronto, ON, M5S 3E1, Canada; ^3^ Department of Chemistry, University of Toronto, Toronto, ON, M5S 3H6, Canada; ^4^ Department of Surgery, University of Toronto, Toronto, ON, M5G 2C4, Canada; ^5^ Li Ka Shing Knowledge Institute, Saint Michael's Hospital, Toronto, ON, M5B 1W8, Canada; ^6^ Department of Laboratory Medicine and Pathobiology, University of Toronto, Toronto, ON, M5S 1A8, Canada; ^7^ McCaig Institute, University of Calgary, Calgary, AB, T2N 4Z6, Canada; ^8^ Department of Cell Biology and Anatomy, Cumming School of Medicine, University of Calgary, Calgary, AB, T2N 4N1, Canada; ^9^ Institute of Cell Biology, Center for Molecular Biology of Inflammation, University Münster, 48149 Münster, Germany; ^10^ Third Institute of Physics – Biophysics, Georg August University Göttingen, 37077 Göttingen, Germany; ^11^ Department of Cell and Systems Biology, University of Toronto, Toronto, ON, M5S 3G5, Canada

**Keywords:** Myoblast, Substrate stiffness, Nuclear envelope, Cell contractility, Extracellular matrix, Mechanobiology

## Abstract

Understanding how biophysical and biochemical microenvironmental cues together influence the regenerative activities of muscle stem cells and their progeny is crucial in strategizing remedies for pathological dysregulation of these cues in aging and disease. In this study, we investigated the cell-level influences of extracellular matrix (ECM) ligands and culture substrate stiffness on primary human myoblast contractility and proliferation within 16 h of plating and found that tethered fibronectin led to stronger stiffness-dependent responses compared to laminin and collagen. A proteome-wide analysis further uncovered cell metabolism, cytoskeletal and nuclear component regulation distinctions between cells cultured on soft and stiff substrates. Interestingly, we found that softer substrates increased the incidence of myoblasts with a wrinkled nucleus, and that the extent of wrinkling could predict Ki67 (also known as MKI67) expression. Nuclear wrinkling and Ki67 expression could be controlled by pharmacological manipulation of cellular contractility, offering a potential cellular mechanism. These results provide new insights into the regulation of human myoblast stiffness-dependent contractility response by ECM ligands and highlight a link between myoblast contractility and proliferation.

## Teaser

**Highlighted Article:** Exploration of the activities of myoblasts over a range of substrate stiffnesses and extracellular matrix components to understand their effects on myoblast contractility, which was found to be linked to proliferation.

## INTRODUCTION

The human skeletal muscle system sustains body mobility for many years owing to its self-repair capacity, powered by muscle stem cells (MuSCs). These MuSCs are scattered throughout muscle tissues in between muscle fibers and exist mostly in a quiescent state during homeostasis. In the event of tissue damage, MuSCs become activated, begin to proliferate, and either fuse into muscle fibers or self-renew. These processes are carefully regulated by biophysical and biochemical cues over the tissue repair period (Li et al., 2018; Loreti and Sacco, 2022). MuSCs reside in a niche that is structurally polarized with the basal lamina on one side and the muscle fiber sarcolemma on the other. The basal lamina is a sheet of extracellular matrix (ECM) composed of laminins, collagens, fibronectin and proteoglycans. During tissue repair, the ECM surrounding MuSCs can be both produced and degraded by many cell types, including MuSCs themselves, causing fluctuations in its composition and physical properties, like stiffness (Loreti and Sacco, 2022). Stiffening of bulk muscle tissue and single myofibers occurs upon damage (Lacraz et al., 2015; Silver et al., 2021; Trensz et al., 2016), aging (Alnaqeeb et al., 1984; Blanpied and Smidt, 1993; Lacraz et al., 2015; Stearns-Reider et al., 2017; Wood et al., 2014), exercise (Green et al., 2012) and disease (Engler et al., 2004; Lacourpaille et al., 2017; 2015; Stedman et al., 1991), whereas engineered niche stiffness has been shown to control myogenic cell activation (Monge et al., 2017), proliferation (Bauer et al., 2017; Boontheekul et al., 2007; Lacraz et al., 2015; Madl et al., 2021; Trensz et al., 2016), differentiation (Boontheekul et al., 2007; Bunney et al., 2017; Engler et al., 2004; Lacraz et al., 2015; Madl et al., 2021; Trensz et al., 2016), self-renewal (Gilbert et al., 2010) and division symmetry (Moyle et al., 2020). However, a less-explored area is the role of specific ECM ligands in regulating the stiffness-sensing of myogenic cells. Given that irregular ECM remodeling is often involved in skeletal muscle aging and disease pathology, it is necessary to extend our understanding of the synergy between the physical and compositional changes in the ECM in controlling myogenic cell behavior.

MuSC adhesion to the ECM is mediated by transmembrane integrin receptors, with the most well-known being integrin α7, who together with the β1 subunit form binding receptors to niche laminin. MuSCs can also express integrins α3 (Brzóska et al., 2006) and α6 (Rayagiri et al., 2018; Wilschut et al., 2011), with MuSCs progenitors *in vitro* expressing more integrin subtypes (Gullberg et al., 1998). Integrin subtypes are specific to the ECM ligands present, therefore any compositional changes in the ECM immediate to the MuSC microenvironment hypothetically would shift the profile of integrin subtypes recruited to the MuSC membrane. For instance, laminin-α1 deposition has been reported to couple with the appearance of integrin α6 during MuSC activation (Rayagiri et al., 2018). Given that integrin subtypes can have differential binding mechanics with the ECM and thus tension-transmitting properties (Seetharaman and Etienne-Manneville, 2018), ECM compositional dynamics could not only alter niche stiffness but potentially also how MuSCs ‘feel’ that change. This hypothesis is challenging to test in the native MuSC niche, because there is limited ability to manipulate physical niche factors without introducing chemicals disturbing the biochemical homeostasis. *In vitro*, murine MuSC commitment to differentiation was reported to be sensitive to substrate stiffness on RGD-presenting hydrogels (RGD is most known as the integrin-binding domain of fibronectin, although it is also present in other ECM proteins), but not on laminin-presenting hydrogels (Madl et al., 2021). MuSC proliferation and migration have also been shown to be controlled by both ECM ligands and substrate stiffness by an unknown mechanism (Boonen et al., 2009; Calve and Simon, 2012; Madl et al., 2021). Deconstructing the interplay between ECM ligands and substrate stiffness converges at two questions – how ECM ligand identity changes the way myogenic cells sense substrate stiffness, and how substrate stiffness changes ECM ligand signaling. In the current work, we contribute to the former question by characterizing the effects of ECM ligands on the early adhesive and contractile response of myoblasts to substrate stiffness, and then further emphasize the relationship between myoblast contractility and proliferation by reporting a correlation between nuclear envelope wrinkling level and Ki67 (also known as MKI67) expression.

## RESULTS

### Accentuated myoblast stiffness sensitivity observed on fibronectin-tethered substrates

We first explored the influence of ECM ligand tethering on human primary myoblast (passage 7–9; donors aged 60–68) responses associated with stiffness-sensing that included cell spreading area, focal adhesion assembly and traction stress. We followed a well-established polyacrylamide (PA) hydrogel fabrication protocol to create cell culture substrates with tuned stiffness that we then covalently tethered with ECM ligands found within the native muscle stem and progenitor cell microenvironment: fibronectin (FN), laminin (LAM) or collagen-1 (COL) (Tse and Engler, 2010). Hydrogel Young's moduli were measured using compression testing (Fig. S1A). We selected PA hydrogels with moduli spanning 2 to 38 kPa to mimic the reported range of skeletal muscle and single muscle fiber stiffnesses in healthy, damaged and aged states (Engler et al., 2004; Gilbert et al., 2010; Lacraz et al., 2015; Moyle et al., 2020; Safaee et al., 2017; Urciuolo et al., 2013).

**Fig. 1.**
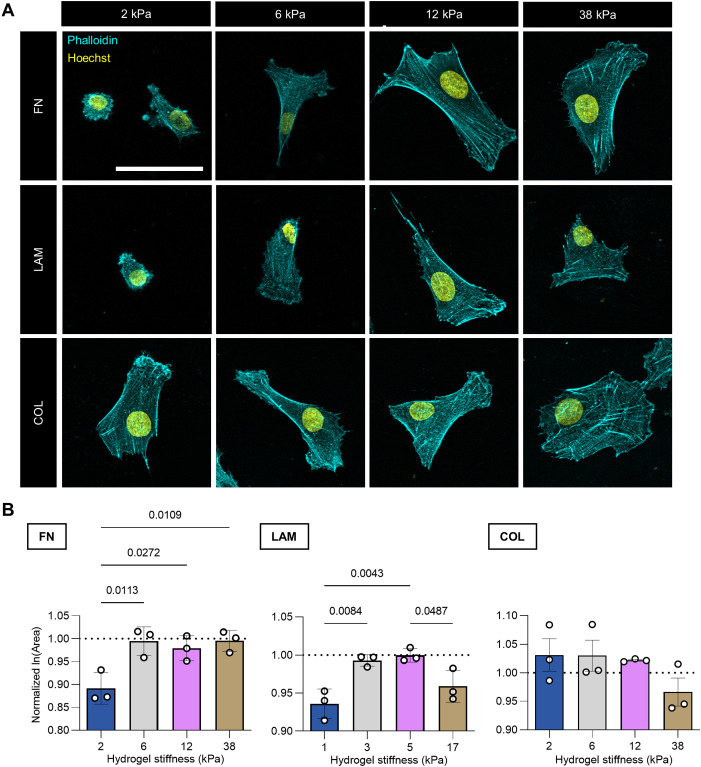
**Cell spreading area characterization across substrate stiffness and ECM conditions.** (A) Representative confocal images of phalloidin staining in primary human myoblasts cultured for 16 h on 1, 3, 5 or 38 kPa polyacrylamide gels tethered with fibronectin (FN), laminin (LAM) or collagen 1 (COL). Phalloidin, cyan; Hoechst, yellow. Scale bar: 50 µm. (B) Graphs showing mean±s.d. cell spreading area across hydrogel stiffness, in FN, LAM, and COL conditions, in terms of fold change with respect to the 14 kPa condition (dotted line at *y*=1). Each data point represents the mean of one biological replicate. *n*=395–920 cells per condition across *N*=3 biological replicates. Statistical comparisons were made by two-way ANOVA followed by Tukey's multiple comparisons tests. See also Table S5.

At 16 h after plating on FN substrates, human primary myoblast cell spreading was minimal on 2 kPa culture substrates, noticeably increased at 6 kPa, then plateaued from 6 to 38 kPa (Fig. 1; Fig. S1B–F). Cell spreading on LAM substrates presented a similar relationship with culture substrate stiffness, although a slight decrease in spreading at 38 kPa was observed. By contrast, cells on COL substrates attained equally large spreading area independently of culture substrate stiffness within the tested stiffness range, except for a decrease at 38 kPa (Fig. 1). As we only included well-isolated mononucleated cells in our analysis, the decrease in spreading area at 38 kPa might have been an analysis artifact due to the observed greater likelihood of larger cells making physical contact with neighboring cells.

**Fig. 2.**
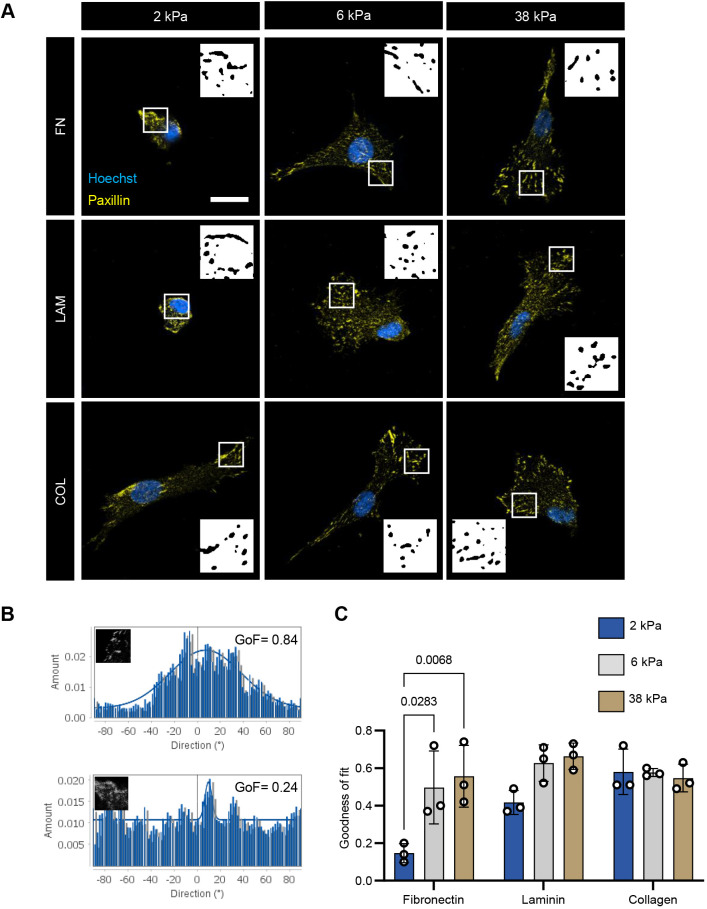
**Adhesion alignment characterization across substrate stiffness and ECM conditions.** (A) Representative confocal images of primary human myoblasts adhesion structures 16 h post-seeding on fibronectin (FN), laminin (LAM) or collagen 1 (COL)-tethered polyacrylamide gels. Paxillin, yellow; Hoechst, blue. Insets are binarized images of paxillin staining in the regions indicated by white boxes. Scale bar: 20 μm. (B) Representative directionality histograms of a paxillin staining ROI (inset) with goodness of fit to a Gaussian curve (GoF)=0.24 (top) and an ROI with GoF=0.84 (bottom). *y*-axis is the frequency of structures detected by the algorithm and *x*-axis is the angle direction of those structures with respect to a horizontal line (°). (C) Bar graph showing GoF as a metric of adhesion alignment. Each data point represents the mean of one biological replicate. *n*=26–32 cells per condition across *N*=3 biological replicates. See also Table S5. Error bars report mean±s.d. Statistical comparisons were made by two-way ANOVA followed by Holm–Šídák multiple comparisons tests.

Next, we visualized adhesion structures with paxillin immunostaining and noted that the paxillin structures detected had a broad range of sizes (Fig. 2A). On the 2 kPa substrates tethered with FN and LAM, wherein the myoblasts had minimal spreading, we observed long paxillin-positive structures outlining the nucleus and/or the peripheral edges of the cells. The appearance of cells with these features was rare on the higher substrate stiffnesses and were also rare on the COL-tethered substrates more generally. Adhesion structure morphologies varied along three scales – within a single cell, across different cells cultured on the same substrates, and between the biological replicate cell lines. These variabilities made it challenging to attribute substrate effects to adhesion size and shape, which, by themselves, are insufficient to infer focal adhesion function and maturity (Geiger et al., 2009). For this reason, we instead chose to quantify focal adhesion structure alignment. Given that cells exert traction forces through adhesion structures where stress fibers terminate, the alignment of adhesion structures is indicative of the alignment of stress fibers and provides a speculative imagery of the cell contractility state (Wu et al., 2012; Zinn et al., 2019). To measure the alignment of the paxillin-positive adhesion structures in confocal images (Fig. 2B, insets), we used the ‘Directionality’ ImageJ plugin, which generates a frequency distribution histogram of structures over a range of direction angles (with 0° being the ‘east’ direction and going counterclockwise). The histogram was then fitted to a Gaussian curve. If the structures in the region of interest align in a common direction, the histogram will have a peak resembling a Gaussian curve with high deviations about the *y*-axis mean, thus having a high goodness of fit (GoF) (Fig. 2B, top). Conversely, if a common direction of alignment does not exist, the histogram will be flat and have a low GoF (Fig. 2B, bottom). The alignment of paxillin structures across substrate conditions (Fig. 2C) showed trends like the cell spreading area data (Fig. 1). Specifically, on FN and LAM substrates, GoF was low at 2 kPa, increased at 6 kPa and then remained relatively constant at 38 kPa, although the increase on LAM substrates was more subtle, and did not reach statistical significance in our study (Fig. 2C). On the COL substrates, adhesion structures reached equally high alignment regardless of substrate stiffness (Fig. 2C).

**Fig. 3.**
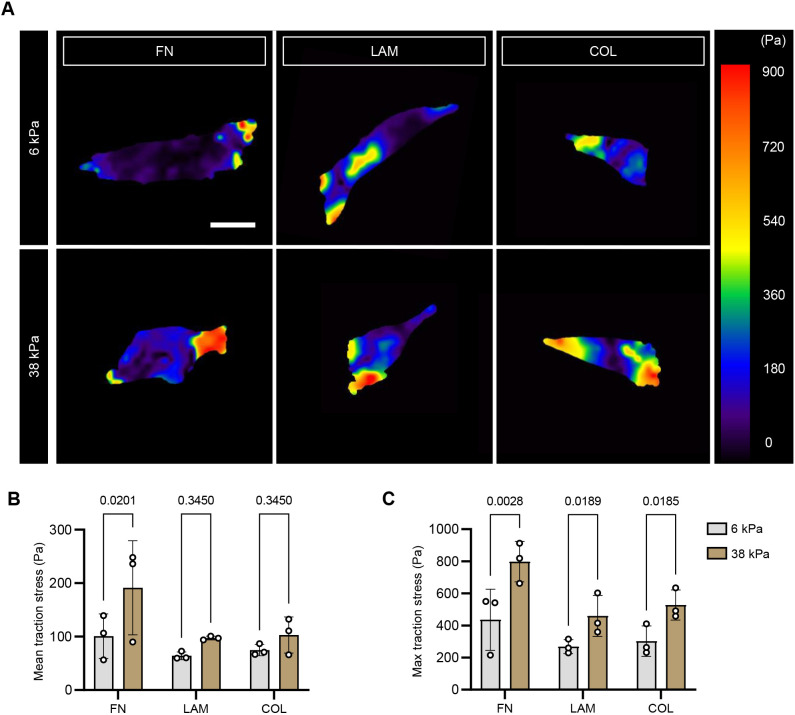
**Cellular traction stress measurement across substrate stiffness and ECM conditions.** (A) Representative traction stress maps of myoblasts 16 h post-seeding on fibronectin (FN), laminin (LAM) or collagen 1 (COL)-tethered polyacrylamide gels. Scale bar: 20 μm. (B,C) Bar graphs showing mean and maximum traction stress, respectively, across substrate stiffness and ECM conditions. *n*=19–24 cells per condition across *N*=3 biological replicates. Each data point represents the mean of one biological replicate. See also Table S5. Error bars report mean±s.d. Statistical comparisons were made by two-way ANOVA followed by a Holm-Šídák multiple comparison test.

Although cell spreading area and adhesion alignment did not significantly differ between 6 and 38 kPa on FN substrates, upon conducting traction force microscopy studies, we observed a significant increase in mean and max traction stress across these conditions. Similar trends were seen on the LAM and COL substrates, though the magnitude of the effect was more nuanced than on the FN condition (Fig. 3A–C). Traction data on 2 kPa substrates were excluded from this analysis because we were unable to reliably derive cellular traction stress with our workflow because of the extremely large hydrogel deformations. Even though lower stresses were recorded on 6 kPa substrates compared to 38 kPa (Fig. 3B,C; likely due to large deformations on soft hydrogels), strain energy was generally higher on 6 kPa than 38 kPa in all ECM conditions, but was only statistically significant for COL (Fig. S2).

In summary, by evaluating the morphology, focal adhesion arrangement and cellular traction stress of primary human myoblasts cultured on 2, 6 and 38 kPa PA hydrogels, we found that FN-tethered hydrogel substrates encouraged more pronounced stiffness sensitivity when compared against the LAM- and COL-tethered substrates, across the metrics tested.

### ECM ligands influence the linearity of proliferation correlation with substrate stiffness

**Fig. 4.**
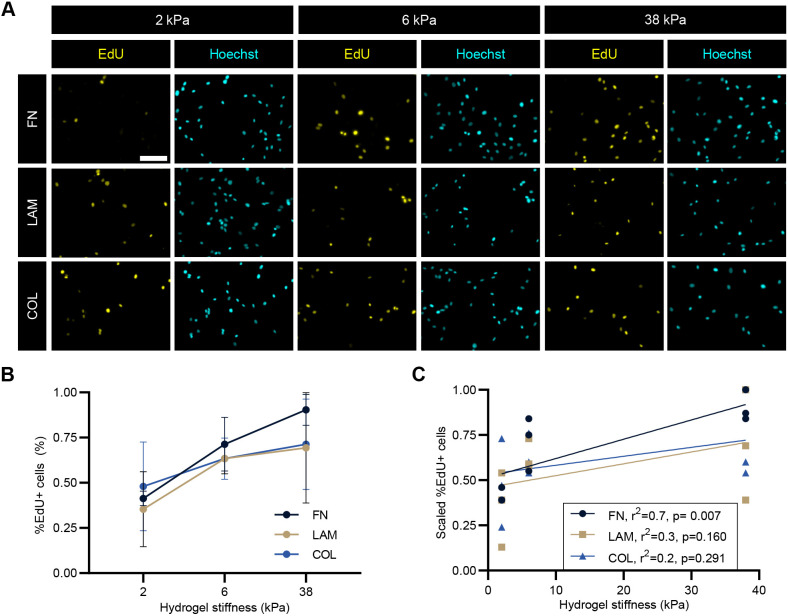
**Myoblast proliferation exhibits a more linear correlation with stiffness on FN substrates.** (A) Representative immunostaining images of EdU incorporation assay at 16 h post-seeding on fibronectin (FN), laminin (LAM) or collagen 1 (COL)-tethered polyacrylamide gels followed by a 12 h EdU pulse. EdU, yellow; Hoechst, cyan. Scale bar: 100 μm. (B) Line graph showing the mean±s.d. of percentage (%) total EdU-positive (+) cells. Statistical comparisons were made by two-way ANOVA followed by Holm–Šídák multiple comparison test. (C) Linear regression analysis of the same data set in B. EdU percentages scaling was conducted by normalizing to the max value of each cell line in order to eliminate cell line variation (scaled *x*=*x*/*x*_max_). Legend displays *r*^2^ (goodness of fit) and *P*-value testing to determine whether the slope of the fitted line is significantly non-zero. Each data point represents the mean of one biological replicate. *n*=462–923 cells per condition across *N*=3 biological replicates. See also Table S5.

To probe whether ECM ligands also tune cellular functional response to substrate stiffness, we evaluated myoblast proliferation using the 5-ethynyl-2′-deoxyuridine (EdU) assay (Fig. 4). After cells were cultured for 16 h on hydrogel substrates, we added EdU in the culture medium for 12 h. The percentage of EdU-positive cells increased steadily from 2 to 38 kPa for myoblasts cultured on FN-tethered substrates (Fig. 4A,B). For LAM- and COL-tethered substrates, the percentage of EdU-positive cells showed an insignificant, but trended increase from 2 to 6 kPa and then remained constant from 6 to 38 kPa. We suspected that myoblast proliferation and substrate stiffness had a positive, linear correlation on FN but not on LAM and COL substrates. To assess linearity, we scaled the percentage EdU-positive values to minimize cell line variance, while maintaining the correlation slope (refer to Materials and Methods), and then performed linear regression fitting (Fig. 4C). On FN substrates, the percentage of EdU-positive cells and substrate stiffness were indeed linearly fitted with an *r*^2^=0.7, whereas LAM and COL were poorly linear with *r*^2^ values of 0.3 and 0.2, respectively. The correlation slope for the FN condition was also significantly non-zero with *P*=0.007, whereas *P*-values for LAM and COL were higher at 0.160 and 0.291 (Fig. 4C). To eliminate the possibility that non-proliferative cells were instead undergoing apoptosis, we performed a live–dead assay, whose result indicated that all cells across all stiffnesses on FN-tethered substrates were calcein-AM positive and propidium iodide negative (Fig. S3).

### Nuclear envelope wrinkling can predict Ki67 expression

**Fig. 5.**
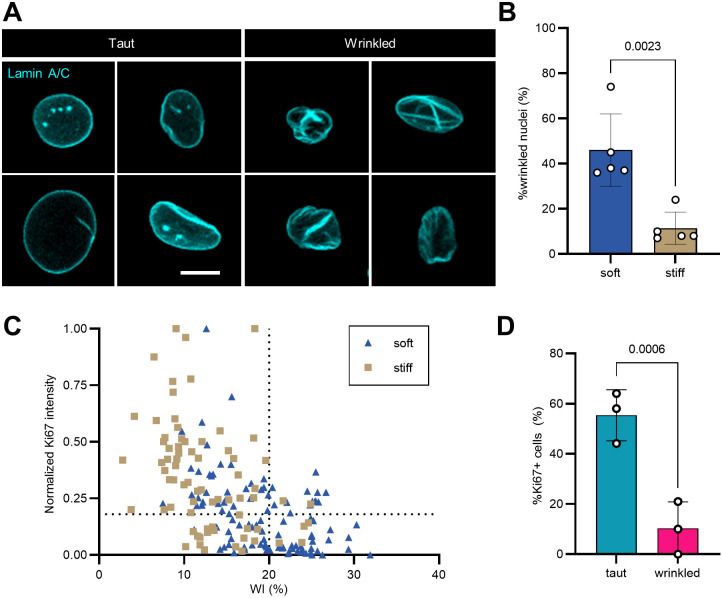
**Stiffness-dependent heterogeneity in nuclear envelope wrinkling predicts Ki67 status.** (A) Representative confocal images of taut [wrinkle index (WI)≤20] and wrinkled (WI>20) nuclear envelopes. Lamin A/C, cyan. Scale bar: 10 μm. (B) Bar graph showing the percentages of taut and wrinkled nuclei on soft and stiff substrates. Each data point represents the mean of one independent technical replicate. Error bars report mean±s.d. *n*=149–121 cells per condition across *N*=5 independent experiments on three different cell lines. See also Table S5. Statistical comparison was made by an unpaired two-tailed *t*-test. (C) *xy* plot with *x*=WI (%) and *y*=normalized Ki67 integrated fluorescence intensity (arbitrary units). Each data point represents one cell. *n*=180 cells total across *N*=3 biological replicates. See also Table S5. The vertical dotted line indicates a threshold above which nuclei were considered wrinkled. The horizontal dotted line indicates a threshold determined by immunofluorescence secondary antibody control below which cells were considered Ki67 negative. (D) Bar graph comparing the percentage of Ki67-positive nuclei in taut and wrinkled nuclei populations. *n*=180 cells total across *N*=3 biological replicates. See also Table S5. Statistical comparison was made by a paired one-tailed *t*-test.

In conducting EdU analysis, we qualitatively noticed that there were more cell nuclei with odd shapes on softer substrates, so we asked whether nuclear envelope (NE) morphology regulation could be upstream of stiffness-dependent proliferation control. We followed through with a series of experiments focusing on two conditions – soft (2 kPa) and stiff (38 kPa) FN substrates because these were the conditions with the most dramatic differences in cellular contractility and proliferation reported in our above results (Figs 1–4). Immunostaining for lamin A/C revealed a heterogeneity in NE shapes on both stiffnesses – some nuclei were taut and oval-like, as generally expected for adherent cells, but some were severely wrinkled (Fig. 5A). We quantified NE wrinkling by measuring wrinkle index (WI) using an ImageJ macro developed by Cosgrove et al. with minor modifications (Cosgrove et al., 2021). We found that taut nuclei with no or minor folding have WI≤20%, whereas wrinkled nuclei have WI>20%. Noticeably, the percentages of wrinkled nuclei, defined by WI>20%, were two times higher on soft compared to stiff FN-tethered substrates (∼40% and ∼19%, respectively) (Fig. 5B). Upon expanding the NE wrinkling analysis to include LAM and COL substrates, we found that the tendency of the nuclei to be less wrinkled on stiff substrates was observed across all conditions but was far less pronounced when compared to the FN substrate conditions (Fig. S4).

We further plotted Ki67 intensity against the WI of each cell in each stiffness condition. Ki67 intensity levels were either negative, low positive (cells in active cell cycle but not S-phase) or high positive (cells in S-phase) (Bruno et al., 1991). We observed that although a taut nucleus did not always have high Ki67 intensity, a nucleus with high Ki67 intensity (normalized Ki67 intensity >0.4) was always taut (WI≤20%), at least across the three cell lines studied (Fig. 5C). Wrinkled nuclei were four times less likely than taut nuclei to be Ki67 positive at any intensity (Fig. 5D).

In summary, there are higher incidences of wrinkled nuclei when myoblasts are cultured on soft substrates, and myoblasts with wrinkled nuclei do not express high levels of Ki67.

### Pharmacological manipulation of contractility modulates NE wrinkling level and myoblast proliferation

**Fig. 6.**
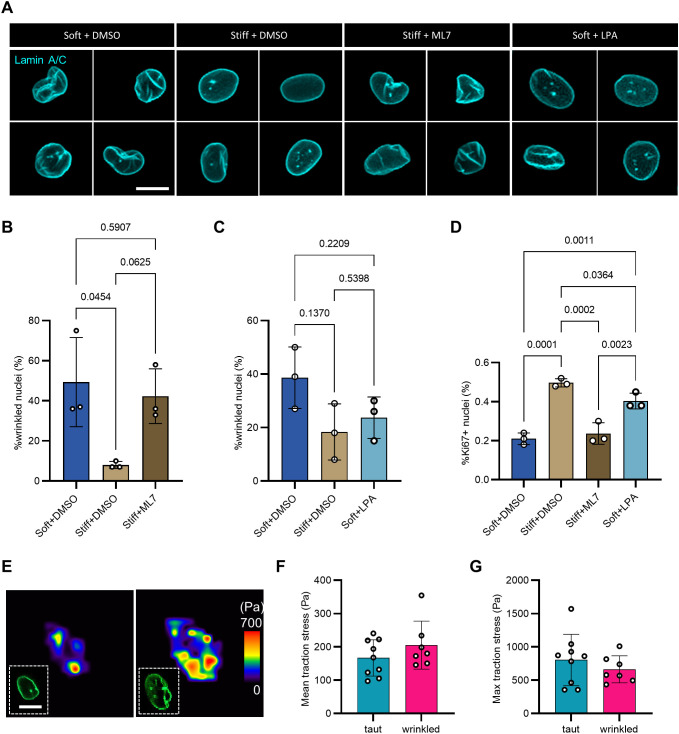
**ML7 and LPA treatments alter myoblast nuclear envelope wrinkling and proliferation.** (A) Representative confocal images of nuclear envelope wrinkling in myoblasts cultured on soft (2 kPa) fibronectin-tethered polyacrylamide substrates treated with either DMSO (soft+DMSO) or LPA for 2 h (soft+LPA), and in myoblasts cultured on stiff (38 kPa) fibronectin-tethered substrate treated with either DMSO (stiff+DMSO) or ML7 1 h before fixing (stiff+ML7). Lamin A/C, cyan. Scale bar: 10 μm. (B) Bar graph showing the percentages of wrinkled nuclei (WI>20) in soft+DMSO, stiff+DMSO, and stiff+ML7 conditions. *n*=74–106 cells per condition across *N*=3 biological replicates. See also Table S5. (C) Bar graph showing the percentages of wrinkled nuclei in the population of myoblasts cultured in soft+DMSO, stiff+DMSO, and soft+LPA conditions. *n*=60–72 cells per condition across *N*=3 biological replicates. See also Table S5. (D) Bar graph showing the percentage of Ki67-positive (+) myoblasts when cells cultured on stiff and soft substrates were treated with, respectively, ML7 and LPA, both at 10 μM overnight, in comparison with DMSO controls. *n*=657–1060 cells across *N*=3 biological replicates. See also Table S5. For all graphs, each data point represents the mean of one biological replicate. Error bars report mean±s.d. Statistical comparison was made by one-way ANOVA followed by a Holm–Šídák multiple comparison test. (E) Representative traction stress maps of a cell with a taut nucleus and a cell with a wrinkled nucleus. Insets show the nuclear envelope of the cells marked by lamin A–GFP reporter. Scale bar: 10 µm. (F,G) Bar graph showing the mean and maximum traction stress of cells with taut and wrinkled nuclei. *n*=7–9 cells in each condition across two technical replicates of one lamin A–GFP transduced cell line. See also Table S5.

Cellular contractile force is transmitted by actin retrograde flow from adhesion sites towards the cell center, where the cytoskeleton is directly linked to the nucleus (Gomes et al., 2005; Swaminathan et al., 2017; Vogel and Sheetz, 2006). To investigate whether low contractility on soft substrates causes NE wrinkling in myoblasts, as has been observed in mesenchymal stromal cells (Cosgrove et al., 2021), we mirrored established methods to pharmacologically inhibit and activate cellular contractility using a myosin light chain kinase inhibitor (ML7) and lysophosphatidic acid (LPA), respectively. The concentrations of ML7 and LPA treatments we used to treat myoblasts were selected based on those used for mesenchymal stromal cells (Cosgrove et al., 2021). Consistent with the mesenchymal stromal cell studies, we found that ML7 treatment increased the percentage of wrinkled nuclei on stiff substrates, whereas LPA treatment lowered the incidence of wrinkled nuclei on soft substrates, although the impact was not as pronounced as ML7 (Fig. 6A–C). Additionally, treatment with ML7 decreased the proportion of Ki67-positive myoblasts present on stiff culture substrates, whereas LPA increased the Ki67-positive proportion on soft substrates (Fig. 6D).

With these results, we hypothesized that cellular traction stresses would be lower in cells with wrinkled nuclei (WI>20) compared to cells with taut nuclei (WI≤20%). To test this hypothesis, we conducted traction force microscopy on myoblasts cultured on 38 kPa FN substrates and stably expressing a lamin A–GFP reporter for NE wrinkling analysis in live cells (Schormann et al., 2020). Unexpectedly, traction stresses and strain energy values were similar between cells with taut and wrinkled nuclei (Fig. 6E–G; Fig. S5). This suggests that the relationship between cellular contractility and NE wrinkling is not straightforward. It is possible that cells with wrinkled nuclei on 2 kPa substrates had lower traction stresses than those on 38 kPa. But if low contractility was the only cause of NE wrinkling, a negative correlation between contractility and WI would have been observed among cells on the same substrate stiffness (Fig. 6E–G; Fig. S5).

In summary, it appears that pharmacological manipulation of cellular contractility can alter NE wrinkling, and the absence of NE wrinkling seems to be a prerequisite for high Ki67 expression in myoblasts. However, low cellular contractility is likely not the only cause of NE wrinkling.

### Proteomic assessment of myoblast cellular processes and fate

To gain a comprehensive understanding of cellular processes characterizing the response to substrate stiffness, we profiled the proteomes of 100,000 cells cultured for 45 h on either 2 or 38 kPa FN-tethered substrates, the timepoints between which we saw the most distinct differences in cellular-level responses. We will refer to these conditions as ‘soft’ and ‘stiff’ substrates in subsequent discussions. 100,000 cells in each stiffness condition were split into three technical replicates. All proteomic data and analysis are provided in the supporting information.

**Fig. 7.**
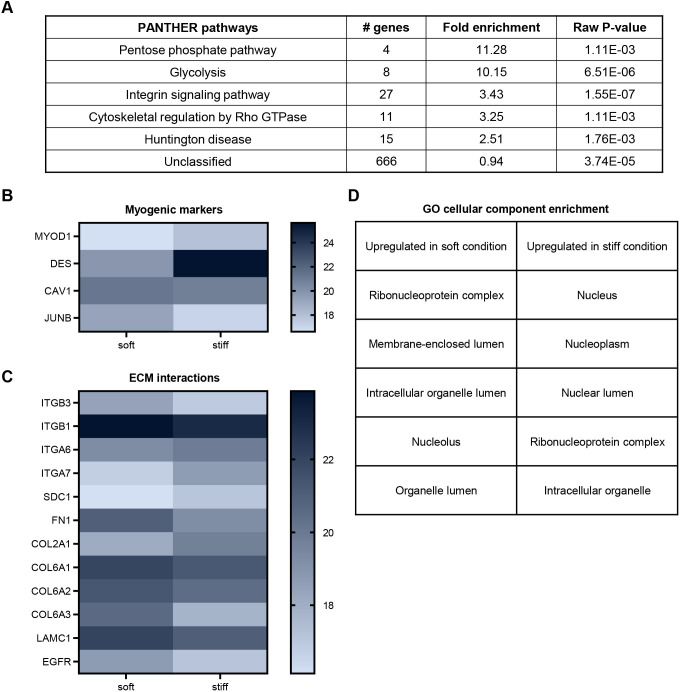
**Profiling myoblast proteomes on fibronectin-tethered soft and stiff culture substrates at 45 h post-seeding.** (A) Overrepresented PANTHER pathways from the list of differentially expressed proteins upon comparing human myoblasts cultured on soft (2 kPa) and stiff (38 kPa) fibronectin-tethered polyacrylamide substrates. The raw *P*-values were determined by PANTHER using Fisher's Exact test or the Binomial statistic. (B) Significant differentially expressed myogenic-relevant markers. (C) Significant differentially expressed proteins with direct ECM interactions. The gradient shade bars in B and C represent relative abundance of proteins detected by mass spectrometry. (D) Table showing top five enriched cellular components from GO cellular component annotation of proteins upregulated in soft and stiff substrate conditions. See also Table S6.

Over 3216 protein groups were detected across the three technical replicates, and only groups with quantitative information in at least two out of the three replicates were considered. Myoblasts cultured on soft and stiff substrates had distinguishable proteomic profiles and among these filtered protein groups, 838 were determined to be differentially expressed by a *P*<0.05 cut-off (Fig. S6A,B). Using the PANTHER database to specifically analyze these differentially expressed proteins, we identified five enriched pathways – the pentose phosphate pathway, glycolysis, integrin signaling pathway, cytoskeletal regulation by Rho GTPase, and Huntington disease (Fig. 7A). The enrichment of integrin signaling and cytoskeletal regulation by Rho GTPase are expected in the context of adherent cell stiffness sensing. Cancer cells cultured on soft environments have been reported to have reduced metabolic activities, which might play a role in decreasing proliferation rate (Tilghman et al., 2012). With the top two overrepresented pathways in our proteomic analysis being pentose phosphate pathway and glycolysis, we further looked at the expression of components of these metabolic pathways, and ATP synthases, for evidence of myoblasts potentially being metabolically dormant on soft substrate. Many enzymes catalyzing chemical processes in pentose phosphate pathway and glycolysis were detected as being more abundant on soft substrates, and ATP synthase subunits expressions had mixed correlation with substrate stiffness (Fig. S6C,D). Without measuring direct metabolic outputs, such as ATP levels, whether there was a metabolic halt on soft substrates remains a standing question, but our proteomic assessment suggests that if there was a halt, it likely would not have been due to the lack of metabolic enzymes or ATP synthase.

Myogenic proliferation and differentiation are regulated by a series of transcription factor activities. *In vitro*, myoblast fate signatures are often profiled by assessing expression of the transcription factors Pax7 and MyoD (also known as MyoD1), with Pax7+/MyoD− being stem-like or reserve-like cells, Pax7+/MyoD+ being proliferative cells, and Pax7−/MyoD+ being differentiating cells. We asked whether there was an increase in the proportion of either reserve-like or differentiating cells that might have corresponded to the larger portion of non-proliferative cells observed on soft culture substrates. We found that the proportions of fate signatures were similar on both stiffnesses, with most cells being Pax7+/MyoD+ (Fig. S7A,B). Interestingly, the proteomics data reported an overall lower MyoD expression for myoblasts cultured on soft substrates (Fig. 7B), whereas desmin, a myogenic commitment marker, was expressed at a higher level on stiff substrate (Fig. 7B). Furthermore, JUNB, which has been previously reported to suppress myogenin expression (Li et al., 1992), was found to express at a higher level on soft substrate (Fig. 7B). Our immunocytochemical analysis suggests that there might not be a major divergence in the Pax7/MyoD myogenic fate landscape across the two substrate stiffness levels at the 45-h time-point post-seeding in growth medium. However, based upon the proteomics assessment, a stiff substrate at 38 kPa would favor a more differentiative phenotype whereas a soft substrate would favor a more reserve-like phenotype. To significantly modulate these fate transitions, we expect that other factors like culture time and medium composition would need to be redesigned.

We noticed higher expressions of ECM proteins like FN1, several COL α-subunits and LAMC1 expressed by cells on soft substrate (Fig. 7C), which could suggest that myoblasts were actively attempting to modify their environment thus not being completely inactive on soft substrate.

We also performed gene ontology (GO) overrepresentation tests on proteins upregulated in either soft or stiff condition. For these tests, we further filtered differentially expressed proteins by their fold change (stiff/soft). Only proteins with fold change less than or equal to 0.9, or more than or equal to 1.1 were considered. Among the top five enriched cellular components in either stiffness were several nuclear components (Fig. 7D). LMNA expression level was found to be higher on a stiff substrate. Given that outside-in environmental signal transduction into cellular function converges at the level of transcription, major changes happening in the nucleus could provide important insights into how substrate stiffness is transduced into a proliferation response.

## DISCUSSION

In this work, we characterized the early time-point ECM ligand-specific cellular responses of passage 7–9 primary human myoblasts (donors aged 60–68) cultured on mechanically tuned polyacrylamide-based hydrogels, described the substrate stiffness-specific proteomes of human myoblasts, and reported that nuclear envelope wrinkling can predict myoblast cell cycle state. Our study revealed that FN ligand tethering led to more exacerbated substrate-stiffness-dependent morphological, contractile and proliferative responses in myoblasts compared to LAM and COL. We also reported that the myoblast proteome responds to stiffness by differentially regulating metabolic, Rho cytoskeletal regulation and integrin signaling pathways, with interesting enrichment of nuclear components. Finally, heavily wrinkled nuclear envelope morphology predicted the absence of Ki67 expression, which was restored by LPA treatment.

The dependence of stiffness-sensing mechanics on ECM ligand is an understudied realm of myogenic cell mechanosensing. ECM ligands bind to specific integrin receptors, which have distinct molecular force transmission mechanics (Seetharaman and Etienne-Manneville, 2018). For example, the FN-integrin α5 bond requires the tension-dependent conformational change of FN to expose a hidden energy site, whereas COL can bind tension independently to integrin α2, leading to substrate stiffness-dependent FAK activation on FN-presenting substrates but not those presenting COL (Seong et al., 2013). The effects of ECM ligands on myogenic cell stiffness-sensing appear to vary depending on the species and passage number of myogenic cells studied and the specific experimental parameters (Boonen et al., 2009; Calve and Simon, 2012; Madl et al., 2021). In our experiments, we observed that relative to LAM and COL, culturing passage 7–9 human myoblasts (donors aged 60–68) on FN-tethered hydrogels led to more exacerbated stiffness-sensitivity in terms of cell spreading, adhesion alignment, cellular traction and proliferation. Whether our results are species, passage or donor age-dependent is an interesting topic for a future study, although the alignment of results between cell lines suggest that donor sex and muscle group do influence the metrics tested in this study. At longer culture time and higher stiffness range, murine MuSCs proliferation have been shown to be stiffness-dependent on both LAM and RGD-presenting hydrogels, whereas MyoG commitment is stiffness-dependent on RGD and not LAM (Madl et al., 2021). Confounding factors to PA hydrogel Young's modulus, such as porosity and ligand tethering density, could potentially add to result variability (Trappmann et al., 2012; Wen et al., 2014). Furthermore, we cannot rule out the possibility that proteins secreted by myoblasts in response to culture within these engineered environments are involved in the cellular responses we report. Regardless, the ECM tethered hydrogels are clearly the stimulatory environment driving the effects we observed. In addition, we want to note that while it was necessary to utilize an *in vitro* system to decouple the effects of various niche factors, the native niche is much more complex. Thus, it is important to acknowledge that in the context of the 3D niche, and the full composition of cellular (vasculature, nerves, immune cells, fibroadipogenic progenitors, etc.), biochemical and physical interactions, the way MuSCs perceive stiffness and the ECM may very well be altered.

The idea that ECM composition could potentially alter myogenic cells mechanosensitivity is important in the context of *in vivo* niche ECM remodeling during regeneration, aging and disease. MuSCs niche factors have been reported to actively suppress activation pathways to maintain MuSCs quiescence (Baghdadi et al., 2018; Zhang et al., 2019). As for LAM, past studies have shown that deficiency in the α2 chain of laminin-2 and laminin-4 leads to the loss of integrin α7β1 (Vachon et al., 1997), which in turn could disrupt quiescence maintenance (Rozo et al., 2016). We can therefore infer that the MuSC connection to LAM via integrin α7β1 is important in preserving MuSC quiescence, but the exact mechanisms are unknown. *In vitro* studies on myoblasts, in agreement with other cell types, showed that LAM preferentially activates Rac and suppresses RhoA (Gu et al., 2001). In contrast with FN, LAM promotes motile cells with diffused stress fibers and poor vinculin-containing adhesion assembly, characteristic of a high Rac and low RhoA phenotype (Goodman et al., 1989). These observations are exciting in the context of the Rac-to-Rho switch occurring during MuSC activation (Kann et al., 2022). Rho-regulated cytoskeletal remodeling is crucial for cell matrix stiffness sensing. It is therefore enticing to explore the possibility that LAM actively suppresses MuSCs mechanosensing, whereas FN enrichment in the injured niche increases MuSC sensitivity to injury sensation and facilitates the quiescent-to-proliferative transition, and furthermore, how age- and disease-related irregular ECM deposition impair MuSC sensitivity to niche remodeling.

MuSCs are functionally heterogeneous (Tierney and Sacco, 2016), as are their downstream progenitors. Our results suggested that relatively stiff substrates, tethered with FN, decrease myoblast cell cycle heterogeneity, cultivating a primarily proliferative population. Although we observed that soft substrates tethered with FN increased the incidence of Ki67-negative myoblasts, we did not assess whether the cell cycle itself was impeded. Regarding the split between Ki67-positive and Ki67-negative myoblasts on the same substrate stiffness, our data suggested that perhaps one of the differences between these subpopulations is their contractility, given that high Ki67 expression was only observed in the absence of NE wrinkling, a state typically characterized by high cellular contractility (Cosgrove et al., 2021). To assess the correlation between cell contractility and NE wrinkling level, we performed traction force microscopy on cells expressing a live lamin A reporter. To our surprise, cellular traction stress and strain energy were independent of the NE wrinkling index, despite pharmacological manipulation of contractility resulting in expected NE wrinkling and Ki67 expression responses. There are a few possible explanations. First, pharmacological manipulation experiments were performed on two substrate elasticities whereas traction force microscopy on lamin A–GFP-expressing cells were conducted exclusively on 38 kPa substrates, so it is possible that cells with wrinkled nuclei on 2 kPa had lower traction stresses than cells with wrinkled nuclei on 38 kPa. Second, our proteomic analysis detected a decrease in lamin A/C on soft substrate, which could potentially take part in increasing NE wrinkling (Buxboim et al., 2017; Kim et al., 2017). Third, we did not assess nuclear actin connectivity. If there is a disruption in tension propagation from the cytoskeleton to the nucleus, nuclear tension would be low regardless of cytoskeletal contractility. For example, severing the linker of nucleoskeleton and cytoskeleton (LINC) complex has been shown to induce similar nuclear wrinkling phenotypes (Cosgrove et al., 2021; Dantas et al., 2022).

Whether and how exactly NE wrinkling could suppress proliferation are questions whose pursuit would require the manipulation of NE wrinkling without altering cellular signaling, which is a limitation of the pharmacological strategies that we and others use to manipulate contractility. LPA activates a range of signaling pathways including Ras and Rho/ROCK GTPases, which are cell cycle regulators (Lin et al., 2010; Manning et al., 1998; Mills and Moolenaar, 2003). Alternatively, low nuclear tension reduces nucleocytoplasmic exchange of signaling molecules like YAP (also known as YAP1), cyclin B1 and MyoD, by potentially deforming nuclear pore complexes (Andreu et al., 2022; Elosegui-Artola et al., 2017; Jacchetti et al., 2021). Cosgrove et al. have reported that there is a correlation between NE wrinkling and YAP/TAZ (TAZ is also known as WWTR1) nuclear localization in mesenchymal stem cells (Cosgrove et al., 2021). In our system, bulk proteomic profiling on soft and stiff substrates did not detect differential YAP target proteins between soft and stiff substrates, suggesting that a YAP/TAZ localization mechanism might not underlie our results.

In conclusion, our work emphasizes the integrated control of substrate stiffness and ECM composition on myoblast contractility and proliferation, which are heterogeneous phenotypes intimately linked to the NE morphology. The impact of our work extends to biomaterial design applications and fundamental knowledge of the regulation of human myogenic cell mechanosensing.

## MATERIALS AND METHODS

### PA hydrogel fabrication

PA hydrogels were fabricated according to the protocol described by Tse and Engler (2010) with some modifications: hydrogels were cast on 35 mm glass bottom dishes (D35-14-1-U, Matsunami Glass, Osaka, Japan) instead of coverslips, and deposition of NaOH was followed by immediate aspiration instead of heated evaporation. Hydrogels were then tethered with either fibronectin (FN; fibronectin bovine plasma, F1141, Sigma, St Louis, MI, USA), collagen (COL; collagen I, rat tail, A1048301, Gibco, Waltham, MA, USA) or laminin (LAM; 11243217001, Roche, Basel, Switzerland) at a 100 mg/ml concentration according the protocol reported by Tse and Engler (2010). Briefly, polymerized hydrogels were covered with sulfo-SANPAH (22589, Thermo Fisher Scientific, Waltham, MA, USA), exposed to UV light for 15 min, and then washed with HEPES buffer three times for 15 min. The hydrogels are then incubated in the ECM proteins overnight at 37°C. Young's moduli of the hydrogel formulations were measured by compression testing using a Mach-1 micromechanical system (Biomomentum, Laval, Quebec, Canada) controlled by a Universal Motion Controller (Newport, Irvine, CA, USA) (Fig. S1A).

For traction force microscopy, PA hydrogels were fabricated following the same protocol above with the addition of embedding 0.1 µm diameter fluorescent beads (F8800, Thermo Fisher Scientific) at a 1:200 dilution.

### Primary human myoblast preparation and expansion

Primary human myoblast cell lines were derived from human skeletal muscle biopsies. Four primary human myoblast cell lines were used in this study: STEM21, STEM38, STEM86 and UCAL38. For the STEM lines, the collection and use of the human skeletal tissues was reviewed and approved by the Providence St. Joseph's and St. Michael's Healthcare Research Ethics Board (REB# 13-370) and the University of Toronto Office of Research Ethics reviewed the approved study and further assigned administrative approval (protocol #30754). Written consent was obtained from donors prior to the scheduled surgical procedure. Human skeletal muscle tissues removed from the multifidus muscle of patients undergoing lumbar spine surgery and designated for disposal were utilized in this study. Recruitment began August 18, 2014 and ended August 18, 2020. For the UCAL38 cell line, the collection and use of cadaveric human skeletal tissues was reviewed and approved by the University of Calgary Research Ethics Board (REB# 15-0005) and the University of Toronto Office of Research Ethics reviewed the approved study and further assigned administrative approval (protocol #37165). Written consent was from next of kin. Recruitment began September 14, 2018 and is ongoing. All procedures in this study were performed in accordance with the guidelines and regulations of the respective Research Ethics Boards, and more broadly, all clinical investigations were conducted according to the principles expressed in the Declaration of Helsinki. Table S1 summarizes the donor information for each line.

The establishment of primary human myoblast cell lines was described in previous reports (Bakooshli et al., 2019; Afshar et al., 2020). To summarize, human skeletal muscle biopsies were minced and incubated with collagenase (Sigma, 630 U/ml) and dispase (Roche, 0.03 U/ml) in Dulbecco's modified Eagle's medium (DMEM; Gibco) to digest the ECM and basal lamina. The resulting cell slurry was then passed through a 20G needle multiple times to obtain a single-cell suspension, followed by incubation with red blood cell lysis buffer (Table S2). The resulting non-purified cells were plated on collagen-coated tissue culture dishes for one passage in myoblast growth medium (Table S2) and then were immunostained and sorted using fluorescence-activated cell sorting (FACS), to purify the CD56+ myogenic progenitor population. These mycoplasma-free (MicoAlert Mycoplasma Kit #LT07-318; Lonza, Cambridge, MA, USA) cell lines are not further authenticated.

Myoblasts were expanded on collagen-coated tissue culture plastic plates before being collected at passage 7–8 for experiments using the following method: adherent cells were first washed with pre-warmed PBS, then incubated in 0.05% trypsin-EDTA (25200072, Thermo Fisher Scientific) at 37°C until all cells detached (∼5 min). After neutralizing trypsin-EDTA with growth medium (see Table S2), the cell solution was centrifuged in 15 ml conical tubes at 300 ***g*** for 8 min, then resuspended in 1 ml fresh growth medium. After cell-counting using a hemocytometer, 20,000 cells were seeded per hydrogel (diameter of 12 mm) for morphology, focal adhesion analysis and traction force microscopy experiments, whereas 25,000 cells were seeded per hydrogel for all other experiments.

### ML7 and LPA treatment

Prior to NE wrinkling analysis, cells were treated with 25 µM ML7 (ab120848, Abcam, Waltham, MA, USA) for 1 h before fixing, or with 50 µM LPA (L7260, Sigma-Aldrich) for 2 h post-seeding, followed by a wash with growth medium before incubating cells in fresh growth medium until the fixation time-point. Prior to Ki67 analysis, treatment with 10 µM ML7 or LPA was conducted overnight. In all of these conditions, the cells were fixed at 45 h post-seeding.

### Immunostaining

Fixation was undertaken by replacing half the volume of medium in the culture dish with 4% paraformaldehyde (15714, Electron Microscopy Sciences, Hatfield, PA, USA) in PBS followed by a 15-min incubation. After three 5-min PBS washes, cells were permeabilized in 0.1% Triton X-100 (TRX777.500, BioShop, Burlington, Ontario, Canada) in PBS for 15 min. Cells were then blocked in 10% goat serum (16210072, Thermo Fisher Scientific) in PBS for 1 h. Immediately after blocking, primary antibodies diluted in 1% goat serum were added and left overnight at 4°C. The following day, after three 5-min washes in 0.025% Tween-20 (TWN510.500, BioShop) in PBS, cells were incubated in secondary antibodies and counterstained with Hoechst 33342 (H3570, Thermo Fisher Scientific) diluted in 1% goat serum for 1 h. For F-actin visualization, Phalloidin was also added at this step. Antibody information and dilution are reported in Table S3. Before imaging, cells were washed for 5 min in 0.0025% Tween-20 in PBS, then a final wash on a gentle shaker for 30 min. All steps were done at room temperature unless specified otherwise. For Pax7 and MyoD experiments, after the final wash, PBS was removed then a coverslip was placed on top of the cells, so that imaging was done through the top coverslip with the plate upside-down in case PA hydrogels with different stiffness alter the perceived fluorescence intensity of the samples, even though this has not been reported as a concern in major PA protocols (Pelham and Wang, 1997; Tse and Engler, 2010). In other experiments, where fluorescence intensity was not crucial, imaging was done through the bottom coverslip of the glass-bottom plate.

### Traction force analysis

After 16 h of culture on PA hydrogels with embedded fluorescent beads (F8800, Thermo Fisher Scientific), the cells were stained for CellTracker Deep Red dye (C34565, Thermo Fisher Scientific) according to the manufacturer's protocol. Imaging was done in FluoroBrite DMEM (A1896701, Thermo Fisher Scientific), 20% FBS (Gibco, 10437028), and 1% penicillin-streptomycin solution medium. Images of beads and cells were taken before and after the addition of 10% SDS (SDS001.500, Bioshop). Traction stress maps were obtained using ImageJ macros developed in prior published work (Martiel et al., 2015; Tseng et al., 2012). Using MATLAB, cell masks were obtained by expanding the original CellTracker binarized image by 70 pixels in all directions. Only traction stress values inside the cell masks were considered.

### EdU assay

After 16 h of culture on hydrogel substrates, cells were pulsed with EdU at a concentration of 1 µM for 12 h. Cells were fixed in 4% PFA for 15 min and incubated in blocking solution containing 10% goat serum and 0.3% Triton X-100 (TRX777, BioShop) diluted in PBS for 45 min at room temperature. EdU immunolabeling was done using the Invitrogen Click-iT EdU kit according to the manufacturer's protocol (C10339, Thermo Fisher Scientific) and then cells were counterstained with Hoechst 33342.

### Viability assay

After 16 h of culture on hydrogel substrates, cells were washed with PBS, then incubated with 1 µM calcein-AM (L3224, Thermo Fisher Scientific), 1 µM propidium iodide (P4864, Sigma) or ethidium homodimer-1 (L3224, Thermo Fisher Scientific), and 1:1000 Hoechst 33342 (H3570, Thermo Fisher Scientific) in PBS for 20 min at room temperature. After removing the staining solution, cells were washed with PBS and imaged immediately.

### Microscopy and image analysis

Except for the EdU analysis, all microscopy was done with a Zeiss Axio Observer 7 microscope equipped with LSM 800 scan head (Zeiss, Oberkochen, Baden-Württemberg, Germany). A Plan-APO 40×/NA 1.40 Oil DIC objective was used for all single cell analyses (i.e. paxillin, lamin A/C, traction force microscopy and phalloidin representative images), and a Plan-Apo 10×/NA 0.45 objective was used for cell spreading area and the Ki67 imaging. EdU staining images were taken using an Olympus IX83 microscope equipped with a DP80 dual CCD camera and either a LUCPLFLN PH 20×/ NA 0.46 or UPLFLN 2PH 10×/ NA 0.3 objective (Olympus, Shinjuku City, Tokyo, Japan).

All image analysis was performed with ImageJ. Focal adhesion alignment by goodness of fit (GoF) to a Gaussian curve was obtained by running the Directionality ImageJ plug-in (written by Jean-Yves Tinevez; https://imagej.net/plugins/directionality) on a random region of interest (ROI) close to the cell periphery on the maximum projection of paxillin immunostaining confocal *z*-stacks. The analysis performed was based on Fourier spectrum analysis. The percentage of total cells labeled by EdU was quantified by dividing the number of segmented objects in the EdU channel by the number of segmented objects in the Hoechst channel. The nuclear wrinkling index was quantified using the ImageJ macro developed by Cosgrove et. al (Cosgrove et al., 2021) with some adjustments to allow semi-batch processing of our datasets. The adapted macro used can be found in our public Github repository (https://github.com/gilbertlabcode/Nguyen2024_JournalofCellScience). Ki67, Pax7 and MyoD fluorescence intensities in each cell were quantified by measuring the integrated density inside each nucleus. Binary binning (+/–) of Ki67, Pax7, and MyoD was done by identifying an intensity threshold below which cells appear ‘negative’ by reference to the secondary antibody controls.

### Lentiviral production

Lentivirus particles were produced by calcium phosphate transfection, as reported previously (Iggo, 2022). Briefly, 35,000/cm^2^ HEK 293 T cells (a gift from Peter Zandstra, Michael Smith laboratories, University of British Columbia, Vancouver, Canada) were seeded in a 10-cm culture plate and co-transfected 2 days later at 80% confluency with second generation lentivirus system. Culture medium (DMEM supplemented with 10% FBS) was refreshed prior to transfection, followed by addition of the plasmid cocktail containing 4 µg of pMD2.G (deposited by Didier Trono; Addgene plasmid #12259), 10 µg of psPAX2 (deposited by Didier Trono; Addgene plasmid #12260) and 15 µg of pLVX-EF1a-GFP-LaminA-IRES-Hygromycin (Schormann et al., 2020; deposited by David Andrews; Addgene plasmid #134867) for 12 h. Culture medium was replaced with OptiMEM (31985062, ThermoFisher Scientific) 20 mM HEPES (51558-50ML, Sigma-Aldrich), and the supernatant containing viral particles was collected at 24 and 48 h after transfection, passed through a 0.45 µm filter, and then concentrated using a 50 ml 100 000 MWCO spin column (Cytiva Life Sciences, Marlborough, MA, USA).

### Lentiviral transduction

Primary human myoblasts were plated in a collagen-coated 6-well plate in growth medium and transduced when the cells were at about 50% confluency (i.e. about 24 h after plating). 150–500 µl of viral supernatant containing 6 µg/ml polybrene (TR-1003-G, MilliporeSigma, Burlington, MA, USA) and was added dropwise to the cells in fresh growth media and then incubated for 12 h. The virus containing medium was aspirated and the cells were returned to regular growth medium for 48 h. The cells were then trypsinized and seeded onto 38 kPa hydrogel substrates for 16 h until traction force microscopy experiment.

### Proteomics

#### Reagents

Trypsin from bovine pancreas (V5113, Promega Biotech, Madison, WI, USA), lysine-C (P8109S, New England BioLabs, Ipswich, MA, USA), protease and phosphatase inhibitor cocktail (78442, Thermo Fisher Scientific), formic acid (FA; F0507-100ML, Sigma-Aldrich), tris(2-carboxyethyl) phosphine (TCEP, C4706-2G, Sigma-Aldrich), iodoacetamide (IAA; I1149-5G, Sigma-Aldrich), acetonitrile (≥ 99.9% ACN, A9551, Thermo Fisher Scientific), and water (W6-4, Thermo Fisher Scientific) were used.

#### Preparation and digestion of protein extracts from myoblasts

Myoblasts cultured on hydrogel substrates for 45 h were collected by incubation with trypLE™ (12604013, Thermo Fisher Scientific). Cell numbers were determined by hemocytometer counting. For each stiffness condition, a total of 100,000 cells were washed three times with cold PBS and resuspended in 20 μl lysis buffer [50 mM PBS, 1% (v/v) protease inhibitor cocktail]. After three continuous freezing and defrost cycles, insoluble portions were separated from the soluble portions by centrifugation at 16,000 ***g*** for 20 min at 4°C. Samples were reduced in 10 mM TCEP (final concentration) at 70°C for 30 min, alkylated with 20 mM IAA (final concentration) in the dark at room temperature for 30 min, followed by incubation with lysine-C (1:60) at 37°C for 1.5 h. Trypsin (1: 25) was then added and incubated at 37°C for 16 h. Finally, digests were quenched by the addition of 1 μl of concentrated formic acid.

#### NanoRPLC-ESI-MS/MS methods

Nanoflow reversed-phase liquid chromatography (NanoRPLC) was performed on an EASY-nLC 1200 ultra-high-pressure system coupled to a Q-Exactive HF-X mass spectrometer equipped with a nano-electrospray ion source (ThermoFisher Scientific). The obtained peptides were automatically loaded onto a C18 trap column (100 μm i.d.×5 cm) and separated by a C18 capillary column (100 μm i.d.×15 cm). The trap column and the analytical column were both packed in-house with 1.9 μm, 120 Å ReproSil-Pur C18 reversed phase particles (Dr. Maisch GmbH). Two mobile phases [A: 0.1% (v/v) FA and B: 80/20/0.1% ACN/water/formic acid (v/v/v)] were used to generate a 130 min gradient with the flow rate of 300 nl/minute (kept at 3% B for 5 min, 90 min from 3% to 30% B, 20 min from 30% to 45% B, 1 min from 45% to 95% B and 14 min to kept at 95% B). Mass spectrometry (MS) was operated in full scan (120,000 FWHM, 400–1100 *m*/*z*) and data-independent acquisition (DIA) modes at positive ion mode. A 25.0 *m*/*z* isolation window was set for MS/MS scan (60,000 FWHM) with stepped HCD collision energy of 25.5%, 27% and 30%. The maximum IT for both MS and MS/MS was 254 ms. The electro-spray voltage was 2.0 kV, and the heated capillary temperature was 275°C. All the mass spectra were recorded with Xcalibur software (version 4.1, Thermo Fisher Scientific).

#### Database search

DIA-NN 1.8 were applied for database searching against the Uniprot_Homo Sapiens database (updated on 11/11/2021, 78120 entries) and the predicted spectrum library. The predicted spectrum library was generated by DIA-NN 1.8 using the same database. The parameters of database searching include up to one missed cleavage allowed for full tryptic digestion, precursor ion mass tolerance and product ion mass tolerance were set 10 and 20 ppm, respectively, carbamidomethylation (C) as a fixed modification, and oxidation(M), acetyl (protein N-term) as variable modifications. Match between runs (MBR) was activated. Peptide spectral matches (PSM) were validated based on q-value at a 1% false discovery rate (FDR).

#### Data processing

Overrepresentation tests were done with PANTHER database version 17.0 released on 2022-02-22 (https://www.pantherdb.org/news/news20220223.jsp) using Fisher's exact test with false discovery rate correction. For PANTHER pathways annotation, the input was a list of differentially regulated proteins in both directions. For GO cellular component annotation, the inputs were upregulated proteins in either soft or stiff conditions.

### Data normalization

For the cell spreading area, cell area data had a log-normal distribution (Fig. S1A,B). To meet the normality assumption underlying the ANOVA test to compare the means between substrate conditions, analysis was done based on the natural log of cell area [ln(Area)]. Additionally, our experiments were repeated on primary human myoblasts lines from independent donors. To minimize cell line variance that could mask substrate effects, we normalized the mean ln(Area) values to that of the values obtained on the 9 kPa condition for each cell line: normalized ln(Area)=ln(Area)/ln(Area)_8kPa_. Pre-normalized data is reported in Fig. S1C–E.

For the EdU assay, aside from reporting the unadjusted data, we scaled each cell line by the maximum percentage total EdU-positive value (scaled×= x/x_max_) before linear regression fitting. Normalization by scaling allowed us to focus on comparing the stiffness-dependent trends and minimized cell line variance in the exact values of the percentage of total EdU-positive cells.

### Statistical analysis

All statistical analysis was conducted using GraphPad Prism 6.0 software. To compare two conditions in experiments with one independent variable, comparisons were done with *t*-tests. To compare more than two conditions in experiments with one independent variable, a one-way ANOVA test preceded Holm–Šídák multiple comparison tests. For experiments with two independent variables, two-way ANOVA preceded Holm–Šídák multiple comparison tests. For all tests, α=0.05. *P*-values used to discuss comparisons between conditions are reported on top of graphs. Experiment replicates and statistical analysis breakdown are reported in Table S4.

### Representative image preparation

Herein we describe alterations that were made in the course of representative image preparation. For Fig. 1, cropped images were placed on a black background separated by white lines. Images were max projection of *z*-stacks. For Fig. 2, raw paxillin staining images were processed according to a published process (Horzum et al., 2014) up to the Log3D filtering step to create representative images. These images were then processed with the Log3D plug-in, binarized, and cropped to create the insets. For Fig. 3, filtered stress maps were created following the process written in the ‘Traction force analysis’ section above. For Fig. 4: The brightness and contrast of raw EdU and Hoechst images were equally adjusted in all conditions. For Fig. 5, individual nuclei were cropped from raw lamin A/C images. The brightness and contrast of Lamin A/C were equally adjusted for all nuclei. For Fig. 6, images from the first three conditions (soft+DMSO, stiff+DMSO, stiff+ML7) were collected from the same experiment so their brightness and contrast were adjusted equally. The images for the soft+LPA condition were collected from a separate experiment, and thus, the brightness and contrast were adjusted to most closely match that of the other conditions. Note that fluorescence intensity was not considered in the analysis of these results.

## Supplementary Material



10.1242/joces.261666_sup1 Supplementary information

Table S5. Compiled raw data.

Table S6. Proteomic Data.
