# Supplementary material for: Culture substrate stiffness impacts human myoblast contractility-dependent proliferation and nuclear envelope wrinkling
Source: J Cell Sci. 2024 Mar 27;137(6):jcs261666. doi: 10.1242/jcs.261666 (PMC11033523; doi:10.1242/jcs.261666)
Supplement: Supplementary information [file joces-137-261666-s1.pdf]

A

Hydrogel formulation %Acrylamide(A)/%Bis-acrylamide(B)	Young's modulus measured by compression testing (kPa)
3%A/0.1%B	2.0 ± 0.3
4%A/0.15%B	5.7 ± 0.4
5%A/0.15%B	12.1 ± 0.6
5%A/0.225%B	14.3 ± 0.6
10%A/0.15%B	37.5 ± 11.4

B

C

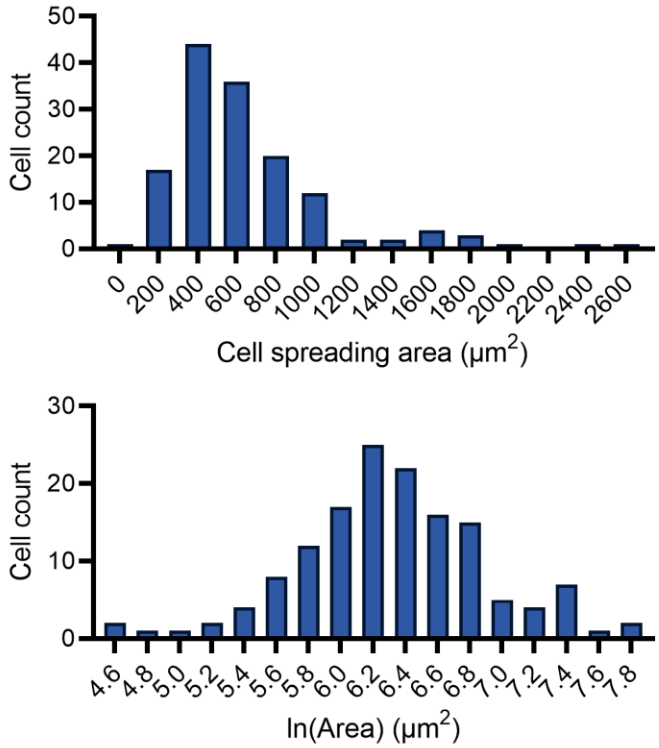

D

E

F

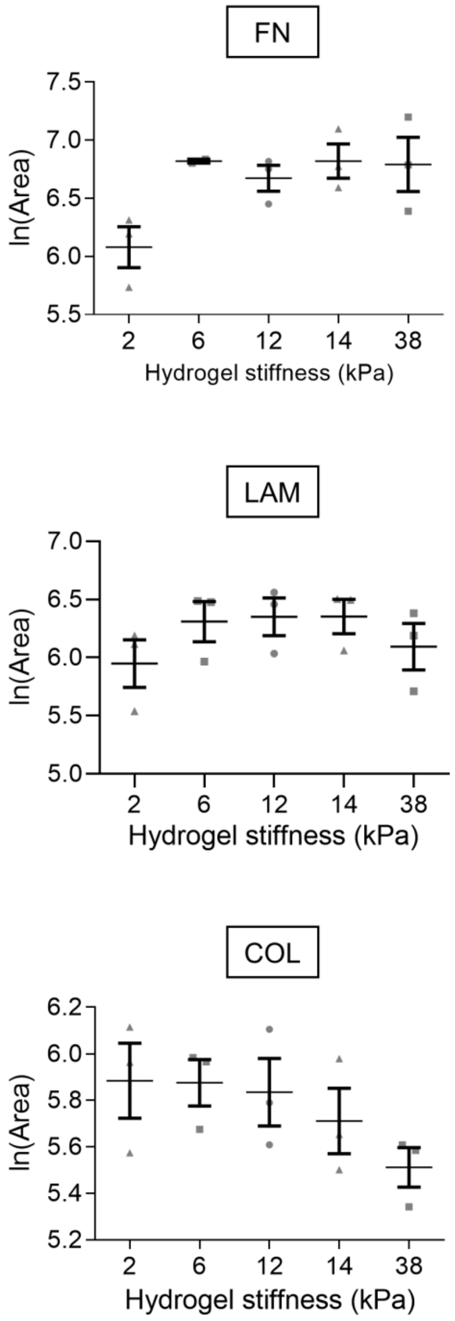

Journal of Cell Science • Supplementary information

Fig. S1. Log-normal cell spreading area distribution A. Young's modulus of each hydrogel's formulation measured by compression testing **B.** Frequency distribution of raw, unadjusted myoblast cell spreading areas on 2 kPa fibronectin-tethered polyacrylamide substrates **C.** Frequency distribution of the natural log of the same cell spreading area data as in B. **D, E, F.** Graphs showing the natural log of myoblast spreading area before normalizing to 14 kPa condition. Each data point represents the mean of one biological replicate. n=395-920 cells per condition across N=3 biological replicates. Error bars report mean ± SD.

Journal of Cell Science • Supplementary information

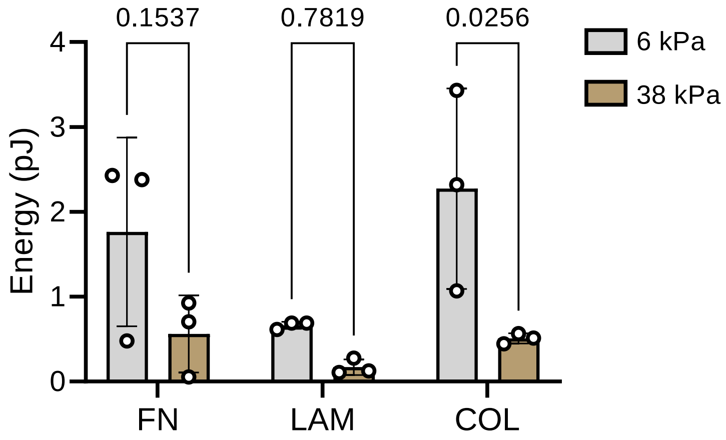

Fig. S2. Strain energy of myoblasts across substrate stiffnesses and ECM culture conditions. Bar graph showing strain energy of myoblasts cultured across substrate stiffness and ECM conditions. n=19-24 cells per condition across N=3 biological replicates. Each data point represents the mean of one biological replicate. Error bars report mean ± SD. Statistical comparisons were made by two-way ANOVA followed by a Holm-Šídák multiple comparison test.

Journal of Cell Science • Supplementary information

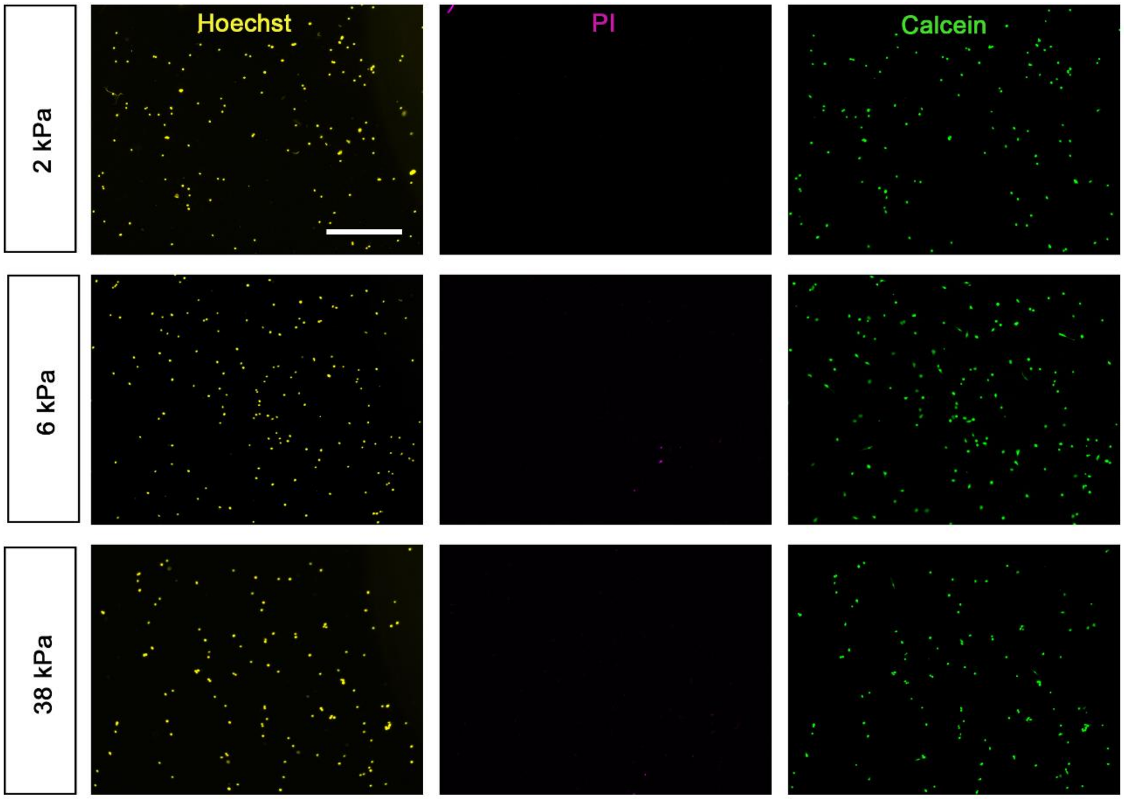

Fig. S3. Viability analysis of myoblasts cultured on fibronectin-tethered polyacrylamide substrates. Representative images of human myoblasts cultured on 2, 6, or 38 kPa fibronectin-tethered polyacrylamide substrates and stained for Hoechst (yellow), propidium iodide (PI; magenta) and Calcein (green). Scale bar=500 μm.

Journal of Cell Science • Supplementary information

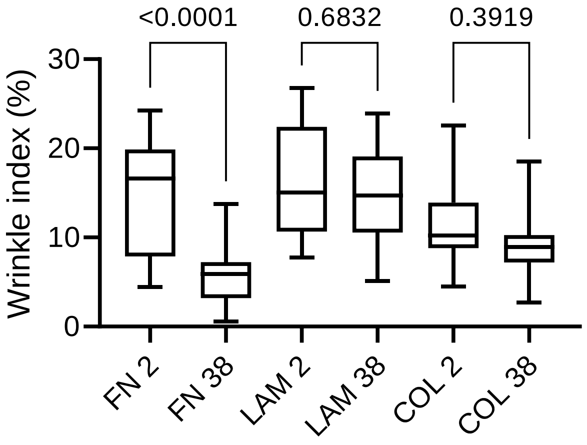

Fig. S4. Nuclear wrinkle index of myoblasts cultured on 2 kPa and 38 kPa substrates tethered with fibronectin (FN), laminin (LAM), and collagen (COL). The whiskers go from minimum to maximum values and the boxes extend from the 25th to the 75th percentile. This is an experiment done with only one cell line. Sample size n is cell number per condition = 21-26. Statistical comparisons were made by ANOVA followed by Holm-Šídák's multiple comparisons tests.

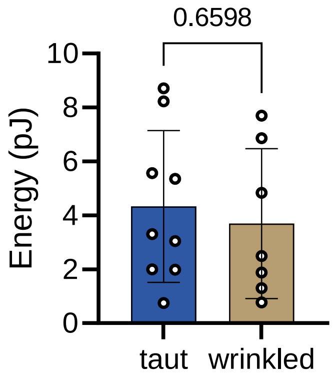

Fig. S5. Strain energy of myoblasts with taut vs. wrinkled nuclei. Bar graph showing strain energy of myoblasts having taut (wrinkle index ≤ 20) vs. wrinkled (wrinkle index > 20) nuclei. n=16 cells in total across 2 technical replicates. Each data point represents one cell. Error bars report mean ± SD. Statistical comparisons were made by an unpaired two-tailed t-test.

Journal of Cell Science • Supplementary information

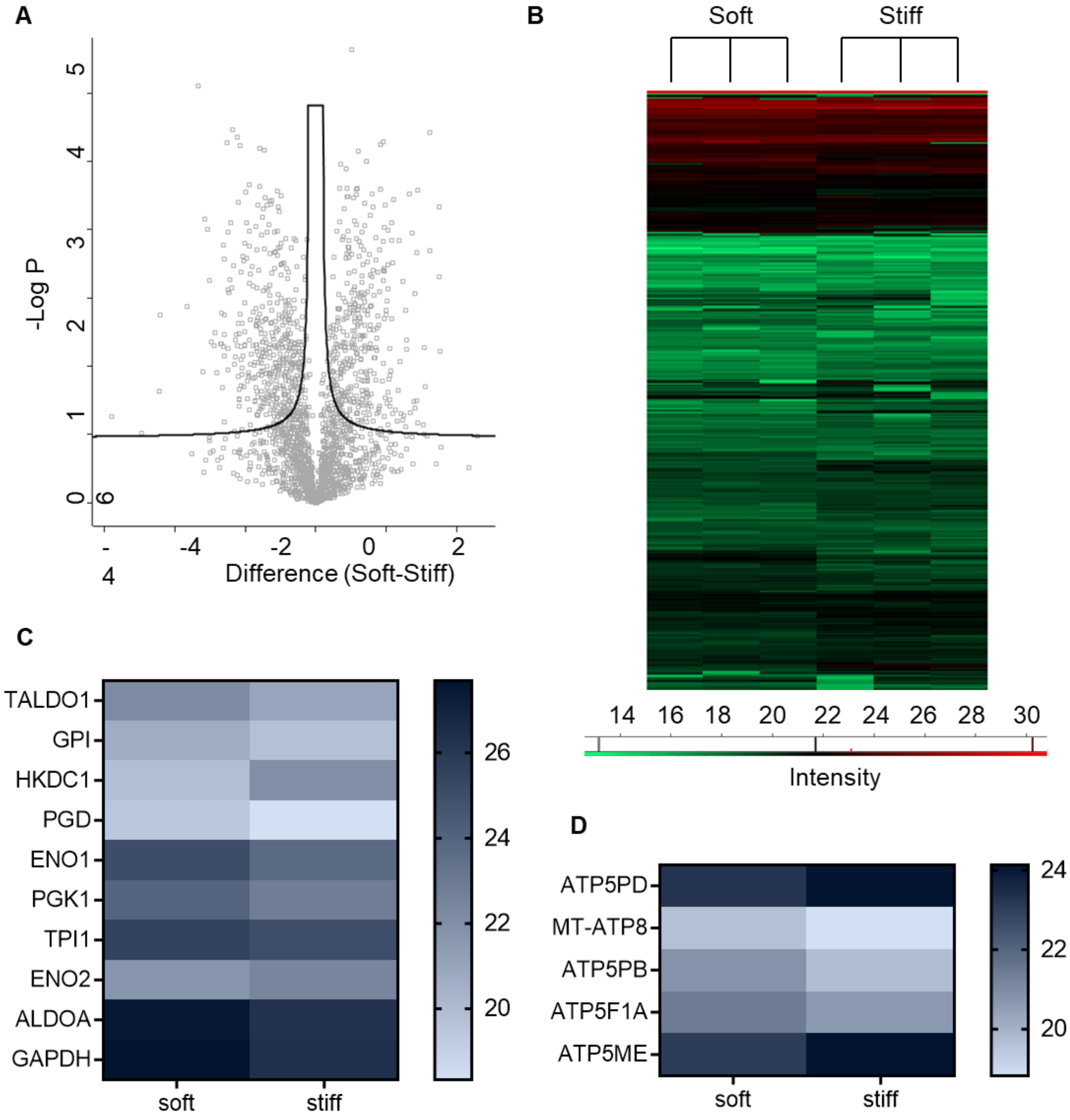

Fig. S6. Proteomic profiling of myoblasts cultured on fibronectin-tethered soft (2 kPa) and stiff (38 kPa) substrates A. Intensity heat map of all protein groups detected in myoblasts cultured on soft and stiff substrate after filtering by the following criteria: at least 2 of the 3 technical replicate runs have quantitative information; and more than 2100 proteins were quantified **B.** Volcano plot showing relative distributions of

upregulated proteins in terms of -Log P-value and expression difference (soft-stiff) of all detected proteins. Proteomic analysis was done on one cell-line with 100,000 cells in each stiffness condition, and split into 3 technical replicates. **C.** Heat map showing protein levels of metabolic enzymes involved in glycolysis and pentose phosphate pathway in soft and stiff substrates **D.** Heat map showing protein levels of ATP synthase subunits on soft and stiff substrates. Scale bars represent relative abundance of proteins detected by mass spectrometry.

Journal of Cell Science • Supplementary information

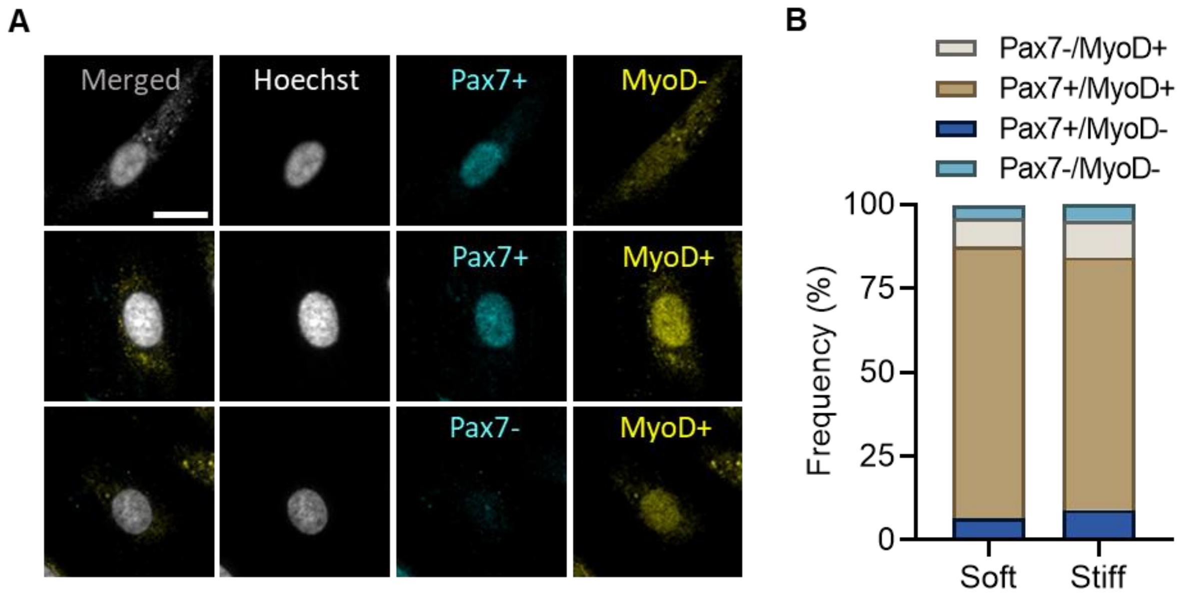

Fig. S7. Pax7/MyoD analysis of myoblasts cultured on fibronectin-tethered soft (2 kPa) and stiff (38 kPa) substrate A. Representative confocal images of myogenic transcription factor immunostaining. Hoechst-grey, Pax7-cyan, MyoD-yellow. Scalebar=20um. **B.** Bar graph showing the percentage of cells in each fate category (Pax7-/MyoD+, Pax7+/MyoD+, Pax7+/MyoD-, Pax7-/MyoD-) on soft and stiff substrate. n=435-469 cells across N=3 biological replicates.

Journal of Cell Science • Supplementary information

Table S1. Skeletal muscle biopsy donor information

Cell line	Donor age	Donor sex	Muscle tissue source
STEM21	64	F	Multifidus muscle of patients undergoing lumbar spine surgery
STEM38	68	M	
STEM86	60	M	
UCAL38	64	M	Gracilis muscle from cadaveric donor within 4 hours of death

Table S2. Cell culture media and solutions

Media and solutions	Composition
RBS lysis buffer	ddH2O, 15.5 mM NH4Cl (Sigma-Aldrich, A9434), 1 mM KHCO3 (Sigma-Aldrich, 237205), 10 µM EDTA
Growth medium	Ham's F-10 Nutrient Mixture (Wisent Bioproducts, 318-050-CL), 20% FBS (Gibco, 10437028), 5 ng/ml fibroblast growth factor-2 (ImmunoTools, 11343625), and 1% penicillin-streptomycin

Journal of Cell Science • Supplementary information

Table S3. Antibody information

Antibody	Host species	Dilution ratio	Manufacturer, catalog number
Hoechst 33342	-	1:1000	Thermo Fisher Scientific, H3570
Fluorescein Phalloidin	-	1:400	Molecular Probes, F432
anti-Paxillin, clone 5H11	mouse IgG1	1:200	Millipore Sigma, 05-417
anti-Pax7	mouse IgG1	1:1	In-house supernatant from hydridoma cell line (DHSB)
anti-MyoD1	mouse IgG2b	1:300	Santa Cruz Biotechnology, sc-377460
anti-Ki67	rabbit	1:300	Abcam, ab16667
anti-Lamin A/C	mouse IgG1	1:300	Santa Cruz Biotechnology, sc-376248
anti-Lamin A/C (H-110)	rabbit	1:300	Santa Cruz Biotechnology, sc-20681
Alexafluor™ 488 Anti-mouse IgG (H+L)	goat	1:300	Invitrogen, A11001
Alexafluor™ 647 Anti-mouse IgG1	goat	1:300	Invitrogen, A21240

Journal of Cell Science • Supplementary information

Alexafluor™ 546 Anti-mouse IgG (H+L)	goat	1:300	Invitrogen, A11003
Alexafluor™ 546 Anti-mouse IgG2b	goat	1:300	Invitrogen, A21141
Alexa Fluor® 647 Mouse Anti-Human CD56 (NCAM-1)	mouse IgG1	1:20	BD Pharmingen, 557711

Journal of Cell Science • Supplementary information

Table S4. Experimental replicates and statistical analysis

Figure	Cell number n and biological replicates N	Sample size n used for statistical analysis and error bars	Statistical analysis
1B	FN 2 kPa: n=515<br>FN 6 kPa: n=395<br>FN 12 kPa: n=548<br>FN 14 kPa: n=594<br>FN 38 kPa: n=601<br>LAM 2 kPa: n=567<br>LAM 6 kPa: n=471<br>LAM 12 kPa: n=414<br>LAM 14 kPa: n=455<br>LAM 38 kPa: n=445<br>COL 2 kPa: n=920<br>COL 6 kPa: n=835<br>COL 12 kPa: n=884<br>COL 14 kPa: n=678<br>COL 38 kPa: n=899<br>from N=3.	n=3 from each condition	two-way ANOVA followed by Tukey's multiple comparison tests
2C	FN 2 kPa: 32<br>FN 6 kPa: 28<br>FN 28 kPa: 26<br>LAM 2 kPa: 30<br>LAM 6 kPa: 30	n=3 from each condition	two-way ANOVA followed by Holm-Šídák multiple comparisons tests

Journal of Cell Science • Supplementary information

	LAM 38 kPa: 30<br>COL 2 kPa: 27<br>COL 6 kPa: 28<br>COL 38 kPa: 29<br>from N=3.		
3B, 3C, S2	FN 6 kPa: 22<br>FN 38 kPa: 20<br>LAM 6 kPa: 19<br>LAM 38 kPa: 21<br>COL 6 kPa: 24<br>COL 38 kPa: 22<br>from N=3.	n=3 from each condition	two-way ANOVA followed by a Holm-Šídák multiple comparison test
4B	FN 2 kPa: 691<br>FN 6 kPa: 923<br>FN 38 kPa: 655<br>LAM 2 kPa: 751<br>LAM 6 kPa: 854<br>LAM 38 kPa: 462<br>COL 2 kPa: 698<br>COL 6 kPa: 828<br>COL 38 kPa: 715<br>from N=3.	n=3 from each condition	two-way ANOVA followed by a Holm-Šídák multiple comparison test
4C	FN 2 kPa: 691<br>FN 6 kPa: 923<br>FN 38 kPa: 655	n=3 from each condition	Linear regression

Journal of Cell Science • Supplementary information

	LAM 2 kPa: 751<br>LAM 6 kPa: 854<br>LAM 38 kPa: 462<br>COL 2 kPa: 698<br>COL 6 kPa: 828<br>COL 38 kPa: 715<br>from N=3.		
5B	Soft: 149<br>Stiff: 121<br>from N=3.	n=5 from each condition	Unpaired two-tail t-test
5C	Soft: 105<br>Stiff: 75<br>from N=3.	N/A	No statistical analysis
5D	Soft: 105<br>Stiff: 75<br>from N=3.	n=3 from each condition	paired one-tail t-test
6B	Soft+DMSO: n=106<br>Stiff+DMSO: n=74<br>Stiff+ML7: n=78<br>from N=3.	n=3 from each condition	one-way ANOVA followed by a Holm-Šídák multiple comparison test
6C	Soft+DMSO: n=60<br>Stiff+DMSO: n=72<br>Soft+LPA: n=63<br>from N=3.	n=3 from each condition	one-way ANOVA followed by a Holm-Šídák multiple comparison test

Journal of Cell Science • Supplementary information

6D	Soft+DMSO: n=1060<br>Stiff+DMSO: n=726<br>Stiff+ML7: n=657<br>Soft+LPA: n=688<br>from N=3.	n=3 from each condition	one-way ANOVA followed by a Holm-Šídák multiple comparison test
S4	FIB 2 kPa: n=22<br>FIB 38 kPa: n=26<br>LAM 2 kPa: n=22<br>LAM 38 kPa: n=21<br>COL 2kPa: n=26<br>COL 38kPa: n=22<br>from N=1	n=cell number	one-way ANOVA followed by Holm-Šídák's multiple comparisons tests.

Table S5. Compiled raw data.

Available for download at
https://journals.biologists.com/jcs/article-lookup/doi/10.1242/jcs.261666#supplementary-data

Table S6. Proteomic Data.

Available for download at
https://journals.biologists.com/jcs/article-lookup/doi/10.1242/jcs.261666#supplementary-data

Journal of Cell Science • Supplementary information
