# Supplementary material for: Culture substrate stiffness impacts human myoblast contractility-dependent proliferation and nuclear envelope wrinkling
Source: J Cell Sci. 2024 Mar 27;137(6):jcs261666. doi: 10.1242/jcs.261666 (PMC11033523; doi:10.1242/jcs.261666)
Supplement: Supplementary file 1 [file joces-137-261666_review_history.pdf]

Culture substrate stiffness impacts human myoblast contractility-dependent proliferation and nuclear envelope wrinkling

Jo Nguyen, Lu Wang, Wen Lei, Yechen Hu, Nitya Gulati, Carolina Chavez-Madero, Henry Ahn, Howard J. Ginsberg, Roman Krawetz, Matthias Brandt, Timo Betz and Penney M. Gilbert
DOI: 10.1242/jcs.261666

Editor: Megan King

Review timeline

Original submission:	27 September 2023
Editorial decision:	3 November 2023
First revision received:	31 January 2024
Accepted:	4 February 2024

Original submission

<u>First decision letter</u>

MS ID#: JOCES/2023/261666

MS TITLE: Culture substrate stiffness impacts human myoblast contractility-dependent proliferation and nuclear envelope wrinkling

AUTHORS: Jo Nguyen, Lu Wang, Wen Lei, Yechen Hu, Nitya Gulati, Carolina Chavez-Madero, Henry Ahn, Howard J Ginsberg, Roman Krawetz, Matthias Brandt, Timo Betz, and Penney M Gilbert
ARTICLE TYPE: Research Article

We have now reached a decision on the above manuscript.

To see the reviewers' reports and a copy of this decision letter, please go to: https://submit-jcs.biologists.org and click on the 'Manuscripts with Decisions' queue in the Author Area. (Corresponding author only has access to reviews.)

As you will see, the reviewers gave favourable reports but raised some critical points that will require amendments to your manuscript. In most cases when they suggest additional experiments there is also an acknowledgement that some may be beyond the scope of this manuscript. While we would encourage you to consider if additional experimental evidence would strengthen the conclusions of your manuscript, at a minimum we expect that additional caveats to the interpretation will be included in the revised manuscript. I hope that you will be able to address these critiques because I would like to be able to accept your paper, depending on further comments from reviewers.

Please ensure that you clearly highlight all changes made in the revised manuscript. Please avoid using 'Tracked changes' in Word files as these are lost in PDF conversion.

I should be grateful if you would also provide a point-by-point response detailing how you have dealt with the points raised by the reviewers in the 'Response to Reviewers' box. Please attend to all of the reviewers' comments. If you do not agree with any of their criticisms or suggestions please explain clearly why this is so.

© 2024. Published by The Company of Biologists under the terms of the Creative Commons Attribution License (https://creativecommons.org/licenses/by/4.0/).

Reviewer 1

Advance summary and potential significance to field

The manuscript by Nguyen et. al. investigates the interactions between ECM based substrate adhesion and substrate stiffness for their impact on muscle progenitor cells. The interactions between adhesion ligands in the ECM and stiffness are important to study as they are modified in pathologic environments when muscle progenitors should be active in regenerating the tissue. The work explores the interaction across a number of assays including cell spreading, focal adhesions and traction forces while also investigating proliferation, nuclear wrinkling and contractility and proteomics. The interrelations of these factors provide novel data showing the ECM substrate does impact stiffness sensitivity to spreading, proliferation, and nuclear envelope wrinkling that is well supported by the data. The manuscript is well written and presented.

Comments for the author

There are a couple of factors that limit the potential impact of the work.
First, not all experiments are carried out and the justification for elimination is not clear. For example, early 8 kPa is used as a common healthy stiffness for muscle, but is not included beyond Fig 1. Also, the nuclear wrinkling is a notable observation, however, it is only examined on fibronectin so that the ECM substrate dependence of wrinkling is not discernable. This could be addressed by justifying the selections made, but would be more complete with additional data particularly on ECM sensitivity of nuclear wrinkling. Second, while the experiments are novel, they are limited to fairly short time periods in growth media. Given potential impact on differentiation as implied in the proteomic data the impact could be amplified if the investigation was carried on through in vitro differentiation to more fully characterize the impact on muscle regeneration.
Minor comments are included below:
Ln 103: It isn't clear what 100 um refers to regarding the fluorescent beads. Is that a diameter or radius? Is so it may be a typo as that would be excessively large for cell-based traction forces.
Ln 143: The drugs ML7 and LPA are not defined upon first usage.
Ln 232: Was there any selection performed on the lentiviral transduced cells or is the assumption that all cells were transduced?
Ln 354: The hypothesis of greater likelihood of large cells making contact with other cells in the stiff matrices is plausible. If the imaging was conducted in an unbiased manner that would seem to be testable based on the images that more cell-cell contacts were present at higher stiffnesses.
Line 372: "high goodness of fit (GoF) (Fig 2B, top)." Should indicate low GoF.
Ln 439: It may be helpful to state if true that the concentrations used here were based on MSCs as it doesn't appear a dose response relationship was established.
Ln 453: It is stated that there is not a correlation between contractility and WI on the same stiffness. However, this data does not appear to be presented.
Line 496: Referenced as Fig 5B, but should be Fig 7B.
Ln 508: The use of fold change is somewhat unclear. Presumable less than 0.9 would be a decreases which is why these proteins would be selected for decreases in response to stiffness. However, in 7A there is a 0.94 fold enrichment that may indicate Log2 fold change and an increase presumably since it is highly significant.
Fig 1: The style of plots for Fig 1B seems to be inconsistent with the rest of the figures which contain bars and color.
Fig 2: The top inset in 2B appears to come from 1 kPa FN, but the lower plot isn't clear. It would seem these would be thresholded as in the images in A in order to determine the angle.
Fig 2: It is not entirely clear how the goodness of fit relates to the alignment. Certainly in 2B the alignment quantified in the lower section is greater, but the residuals from the fit line which would be expected to relate to the goodness of fit, are not appreciably different.
Fig 4: It isn't clear why both subfigures B and C are needed. The scaling is somewhat unclear as only one dot appears at 1.0 where it would be expected that each cell line would have a datapoint at 1.
Fig 5: It appears that all nuclei are considered taut or wrinkled such that the %wrinkled completely dependent on the %taut (and visa versa). Thus, in 5B it would be more straightforward to only plot %wrinkled and would also align more with the text.

© 2024. Published by The Company of Biologists under the terms of the Creative Commons Attribution License (https://creativecommons.org/licenses/by/4.0/).

Fig 5: In 5C it is not clear if the normalized intensity of Ki67 is mean or integrated intensity. It may also be helpful to label the thresholds for low and high positive cells as done with the WI threshold for taut vs. wrinkled.
Fig 5D: The colors here may be misleading as my understanding is that this is %Ki67+ regardless of soft or stiff, but depending on taut or wrinkled. However elsewhere blue is associated with soft and gold with stiff.
Fig 7B-D: The meaning of the scalebars is not provided. Unless they are all significant it could be helpful to designate which differences are significant and beyond threshold in these select gene lists.

Reviewer 2

Advance summary and potential significance to field

The manuscript entitled "Culture substrate stiffness impacts human myoblast contractility-dependent proliferation and nuclear envelope wrinkling" investigates how extracellular matrix ligands and substrate stiffness influence the contractility and proliferation of human myoblasts. The results suggest that fibronectin has a more pronounced impact on myoblast behavior, and there is a connection between myoblast contractility and proliferation. Additionally, softer substrates are associated with changes in nuclear morphology and proliferation markers. These results provide new insights into the regulation of myoblast behavior and offer potential avenues for further research in the context of muscle regeneration and tissue repair.

Comments for the author

Overall, the manuscript is well written, and the experiments are logically organized. However, there are some major and minor concerns that need to be addressed before being suitable for publication.

The major limitation of the study is that all of the analyses were done on aged (60–68 years old, both male and female) human myoblasts. It has been well documented that myogenic responses decline with age, and responses to ECM components could be affected by the chronological age of these cells. In addition, it is not clear why passage 7-8 cells were used in the study. Given that three samples were from patients undergoing lumbar surgery, senescence-associated secretory phenotype (SASP) could play a role. Furthermore, one sample from a cadevaric donor was from a different muscle (multifidas vs. gracilis). Most of the analyses were done on 3 cell lines instead of 4, which one are they? Either way, there is an issue with mixed genders or mixed muscle types. Why not use an animal model and validate the results with human samples?

Second, you can't just assume that a hydrogel formulation would give a similar range of stiffness. Young's modulus should be measured with a rheometer for a specific hydrogel formulation.
1, 3, 5, and 8 kPa are too close in range, and the cellular response could be completely different.

Third, tethering of ECM protein is the most critical method in the study. This should be better explained and tested. How are these proteins being tethered? Over time, are these proteins being changed? Do myoblasts deposit ECM components?

Finally, authors should discuss the role of FAPs and other key niche components of muscle stem cell. There are clear limitations of the study (i.e., oxygen tension, lack of innervation, and Ca2+ regulations), this should be addressed in the discussion section.

First revision

Author response to reviewers' comments

© 2024. Published by The Company of Biologists under the terms of the Creative Commons Attribution License (https://creativecommons.org/licenses/by/4.0/).

Author Response: We would like to thank both reviewers for your time and commitment to improving the quality of our submission!

Reviewer 1 Advance Summary and Potential Significance to Field:
The manuscript by Nguyen et. al. investigates the interactions between ECM based substrate adhesion and substrate stiffness for their impact on muscle progenitor cells. The interactions between adhesion ligands in the ECM and stiffness are important to study as they are modified in pathologic environments when muscle progenitors should be active in regenerating the tissue. The work explores the interaction across a number of assays including cell spreading, focal adhesions, and traction forces while also investigating proliferation, nuclear wrinkling, and contractility and proteomics. The interrelations of these factors provide novel data showing the ECM substrate does impact stiffness sensitivity to spreading, proliferation, and nuclear envelope wrinkling that is well supported by the data. The manuscript is well written and presented.

Reviewer 1 Comments for the Author:
There are a couple of factors that limit the potential impact of the work.
First, not all experiments are carried out and the justification for elimination is not clear. For example, early 8 kPa is used as a common healthy stiffness for muscle, but is not included beyond Fig 1. Also, the nuclear wrinkling is a notable observation, however, it is only examined on fibronectin so that the ECM substrate dependence of wrinkling is not discernable. This could be addressed by justifying the selections made, but would be more complete with additional data particularly on ECM sensitivity of nuclear wrinkling.

Author Response: Downstream of our contractility characterizations, we selected to follow-up on the conditions where we observed the most dramatic differences (i.e. fibronectin tethered to 2 and 38 kPa hydrogels) for the subsequent NE wrinkling analysis. We have now added this justification in our text. We also added new NE wrinkling analysis across 3 ECMs which can be found in Supplementary Figure S4. Consistent with our cell spreading area, focal adhesion alignment and traction force studies, we observed that the most dramatic substrate stiffness dependent changes in NE wrinkling occurred in the context of fibronectin tethered substrates.

Second, while the experiments are novel, they are limited to fairly short time periods in growth media. Given potential impact on differentiation as implied in the proteomic data the impact could be amplified if the investigation was carried on through in vitro differentiation to more fully characterize the impact on muscle regeneration.

Author Response: Our original focus was on short-timepoint myoblast adhesion and contractility because we were interested in how myoblasts physically sense the substrate and how that is linked to the transit amplification stage of regeneration (ie. using myoblasts as model), rather than the later stages of regeneration when differentiation dominates. As noted by the reviewer, our proteomics data pointed to influences of substrate/ECM on differentiation state, which could be studied by evaluating later timepoint. However, given that culturing myoblasts on different substrate stiffness and ECM ligands, and then evaluating cell fate progression has been reported by other studies (https://www.sciencedirect.com/science/article/pii/S014296122100329X#; https://journals.physiology.org/doi/full/10.1152/ajpcell.00015.2009), we felt that aspect offered limited novelty. Instead, with our study, we offer a potential mechanism for proliferation control via contractility.

Minor comments are included below:

Ln 103: It isn't clear what 100 um refers to regarding the fluorescent beads. Is that a diameter or radius? Is so it may be a typo as that would be excessively large for cell-based traction forces.
Author Response: Indeed a typo. It should be 100 nm = 0.1um in diameter. It has been edited.

Ln 143: The drugs ML7 and LPA are not defined upon first usage.
Author Response: Edited

Ln 232: Was there any selection performed on the lentiviral transduced cells or is the assumption that all cells were transduced?

© 2024. Published by The Company of Biologists under the terms of the Creative Commons Attribution License (https://creativecommons.org/licenses/by/4.0/).

Author Response: While not all cells were transduced, only those cells found to express the Lamin A reporter could be included in our study. This is because traction force measurements are conducted on live (rather than fixed) cells, so to co-relate nuclear envelope wrinkling and AFM measurements, at the single cell level, we used the Lamin A-GFP reporter signal. We have updated the Results to make this point more clear.

Ln 354: The hypothesis of greater likelihood of large cells making contact with other cells in the stiff matrices is plausible. If the imaging was conducted in an unbiased manner that would seem to be testable based on the images that more cell-cell contacts were present at higher stiffnesses.

Author Response: The imaging was conducted in an unbiased manner, but big interconnected clumps of cells were often not fully captured within a field of view, which would result in a biased analysis of this particular hypothesis using the datasets we have available. Also, it is often challenging to know where a cell boundary ends, which introduces a secondary analysis issue. As such we prefer to speculate on this point in the Discussion and leave a deeper analysis for another focused study.

Line 372: "high goodness of fit (GoF) (Fig 2B, top)." Should indicate low GoF.
Author Response: Edited

Ln 439: It may be helpful to state if true that the concentrations used here were based on MSCs as it doesn't appear a dose response relationship was established.
Author Response: Edited

Ln 453: It is stated that there is not a correlation between contractility and WI on the same stiffness. However, this data does not appear to be presented.
Author Response: The data corresponding to this statement can be found in Fig 6 E-G and S4, which are referenced within the paragraph where these logic statements are discussed. For clarity, we now include reference to these Figures immediately after this statement.

Line 496: Referenced as Fig 5B, but should be Fig 7B.
Author Response: Edited

Ln 508: The use of fold change is somewhat unclear. Presumable less than 0.9 would be a decreases which is why these proteins would be selected for decreases in response to stiffness. However, in 7A there is a 0.94 fold enrichment that may indicate Log2 fold change and an increase presumably since it is highly significant.

Author Response: The fold change described in line 508 (pre-revised text line number) is protein expression on stiff divided by soft substrate used to further narrow down a list of proteins to be analyzed with PANTHER pathway analysis. The fold enrichment in figure 7A is given by PANTHER as the number of proteins on our list divided by the number of expected proteins in each pathway. We have updated the text to make it clearer that these are two different metrics that are quantified using two different approaches.

Fig 1: The style of plots for Fig 1B seems to be inconsistent with the rest of the figures which contain bars and color.
Author Response: We have updated the style of the plot in Figure 1B to harmonize with those in the rest of the manuscript.

Fig 2: The top inset in 2B appears to come from 1 kPa FN, but the lower plot isn't clear. It would seem these would be thresholded as in the images in A in order to determine the angle.
Author Response: Angles were calculated from confocal images of Paxillin immunostaining signals, as shown in the inset images. We have edited the Methods and Results to make this point more clear.

Fig 2: It is not entirely clear how the goodness of fit relates to the alignment. Certainly in 2B the alignment quantified in the lower section is greater, but the residuals from the fit line which would be expected to relate to the goodness of fit, are not appreciably different.

© 2024. Published by The Company of Biologists under the terms of the Creative Commons Attribution License (https://creativecommons.org/licenses/by/4.0/).

Author Response: In addition to the residuals from the fit line, Goodness of Fit also takes into consideration deviations about the y-axis mean. Deviations about the y-axis mean in the top graph are larger than those in the bottom graph, leading to a higher goodness of fit. We have edited the text to make this point more clear.

Fig 4: It isn't clear why both subfigures B and C are needed. The scaling is somewhat unclear as only one dot appears at 1.0 where it would be expected that each cell line would have a datapoint at 1.

Author Response: In 4B we want to show the data without any scaling, which then makes clear that the error bars are some times quite large due to cell line and experimental variations. In 4C we are showing the scaled data where we can compute the r-squared to report a more linear relationship between %EdU+ and gel stiffness with fibronectin. There are indeed 3 dots at y=1 for each cell line, but they overlap. By zooming in, it is possible to see the individual overlapping points.

Fig 5: It appears that all nuclei are considered taut or wrinkled such that the %wrinkled completely dependent on the %taut (and visa versa). Thus, in 5B it would be more straightforward to only plot %wrinkled and would also align more with the text.
Author Response: Edited. Thank you for this suggestion!

Fig 5: In 5C it is not clear if the normalized intensity of Ki67 is mean or integrated intensity. It may also be helpful to label the thresholds for low and high positive cells as done with the WI threshold for taut vs. wrinkled.
Author Response: We have now updated the graph to include threshold lines used to determine negative and positive Ki67 intensity, and in the Results text indicate the threshold for low/high Ki67 intensity.

Fig 5D: The colors here may be misleading as my understanding is that this is %Ki67+ regardless of soft or stiff, but depending on taut or wrinkled. However, elsewhere blue is associated with soft and gold with stiff.
Author Response: Thank you for this suggestion. We have altered the color scheme for Figure 5D in the revised submission.

Fig 7B-D: The meaning of the scalebars is not provided. Unless they are all significant it could be helpful to designate which differences are significant and beyond threshold in these select gene lists.
Author Response: We have now added a scale bar description and we have edited the legend to specify that all proteins reported in this figure are those where expression was statistically significantly different.

*****

Reviewer 2 Advance Summary and Potential Significance to Field:
The manuscript entitled "Culture substrate stiffness impacts human myoblast contractility-dependent proliferation and nuclear envelope wrinkling" investigates how extracellular matrix ligands and substrate stiffness influence the contractility and proliferation of human myoblasts. The results suggest that fibronectin has a more pronounced impact on myoblast behavior, and there is a connection between myoblast contractility and proliferation. Additionally, softer substrates are associated with changes in nuclear morphology and proliferation markers. These results provide new insights into the regulation of myoblast behavior and offer potential avenues for further research in the context of muscle regeneration and tissue repair.

Reviewer 2 Comments for the Author:
Overall, the manuscript is well written, and the experiments are logically organized. However, there are some major and minor concerns that need to be addressed before being suitable for publication.

The major limitation of the study is that all of the analyses were done on aged (60–68 years old, both male and female) human myoblasts. It has been well documented that myogenic responses decline with age, and responses to ECM components could be affected by the chronological age of

© 2024. Published by The Company of Biologists under the terms of the Creative Commons Attribution License (https://creativecommons.org/licenses/by/4.0/).

these cells. In addition, it is not clear why passage 7-8 cells were used in the study. Given that three samples were from patients undergoing lumbar surgery, senescence-associated secretory phenotype (SASP) could play a role. Furthermore, one sample from a cadevaric donor was from a different muscle (multifidas vs. gracilis). Most of the analyses were done on 3 cell lines instead of 4, which one are they? Either way, there is an issue with mixed genders or mixed muscle types.

Author Response: We acknowledge that our results must be taken in the context of the age of the donors and passage number of the cell lines. We have made clear this aspect of our experimental design in the Methods, and it is now noted in the Results. We have also extended our Discussion to make clear that our conclusions are specific to p7-9 myoblast cells from human donors in the 60-68 years age range. Since our phenotypes were reproducible, our conclusion is that sex and muscle group were not dominant contributors to the phenotypes we report. We now make this point clearer in our Discussion. We have updated the raw data excel file in the Supplementary materials to make clear which cell lines have been used in each study.

Why not use an animal model and validate the results with human samples?
Author Response: Introducing the variable of species was outside of the focus of this specific study. Also, if the animal model data does not mirror human cell behavior, it does not necessarily invalidate either results as it could instead reflect species-specific differences.

Second, you can't just assume that a hydrogel formulation would give a similar range of stiffness. Young's modulus should be measured with a rheometer for a specific hydrogel formulation. 1, 3, 5, and 8 kPa are too close in range, and the cellular response could be completely different.

Author Response: We agree with the reviewer and have conducted new analyses of our hydrogels to address this point. Young's moduli measurements for the hydrogel formulations used in our study were determined from compression testing analysis and the values are updated throughout the manuscript.

Third, tethering of ECM protein is the most critical method in the study. This should be better explained and tested. How are these proteins being tethered? Over time, are these proteins being changed? Do myoblasts deposit ECM components?

Author Response: We have updated the Methods to provide a brief summary of the published ECM tethering protocol we employed. The sulfo-SANPAH crosslinker is fully washed out following the ECM tethering step. Thus, if new ECM is deposited, it would not be covalently bound to the polyacrylamide surface. There is of course the possibility of non-covalent attachments, and this cannot be precluded by our study method. Having said that, in a study focused on decoupling cellular influences of substrate stiffness, porosity & ECM density in the context of the exact same PA hydrogel system used for our study, it was shown that porosity and ECM tethering density were not the dominant factors controlling stromal cell differentiation and contractility (https://www.nature.com/articles/nmat4051). These were also the biological areas of interest in our experiments.
Ultimately the routinely used methods to measure protein tethering, over time, are non-conclusive as the resolution is too low. To really characterize the nature of protein tethering and their time-dependent changes, major studies on this topic, including studies of the PA culture platform we implemented, have used a fibronectin FRET sensor developed by the Viola Vogel lab (for e.g. https://www.nature.com/articles/nmat3339, https://www.nature.com/articles/ncomms9026, https://www.nature.com/articles/nmat4051). It is clear from the differing conclusions of these studies that this remains an open area of inquiry. And that is just for fibronectin. For our study that would mean expanding on collagen and laminin testing, which we hope the reviewer agrees is beyond the scope of this current work.
While it is beyond the scope of this humble study to conduct fibronectin FRET sensor temporal studies and to develop collagen and laminin FRET sensors to quantify the tethering of these molecules, we recognize the need to extend our Discussion to include a discourse on these topics, and have done so accordingly.

Finally, authors should discuss the role of FAPs and other key niche components of muscle stem cell. There are clear limitations of the study (i.e., oxygen tension, lack of innervation, and Ca2+ regulations), this should be addressed in the discussion section.

© 2024. Published by The Company of Biologists under the terms of the Creative Commons Attribution License (https://creativecommons.org/licenses/by/4.0/).

Author Response: We agree with the reviewer's standpoint and have included a new paragraph in the Discussion to expand on limitations of the study.

Second decision letter

MS ID#: JOCES/2023/261666

MS TITLE: Culture substrate stiffness impacts human myoblast contractility-dependent proliferation and nuclear envelope wrinkling

AUTHORS: Jo Nguyen, Lu Wang, Wen Lei, Yechen Hu, Nitya Gulati, Carolina Chavez-Madero, Henry Ahn, Howard J Ginsberg, Roman Krawetz, Matthias Brandt, Timo Betz, and Penney M Gilbert
ARTICLE TYPE: Research Article

I am happy to tell you that your manuscript has been accepted for publication in Journal of Cell Science, pending standard ethics checks.

© 2024. Published by The Company of Biologists under the terms of the Creative Commons Attribution License (https://creativecommons.org/licenses/by/4.0/).
